# Adaptive Robotic Grippers for Intelligent Manipulation: A Review of Structural Compliance, Sensing, and Control

**DOI:** 10.3390/s26196134

**Published:** 2026-09-28

**Authors:** Ruibing Fan, Xingwei Wang, Guowei Shao, Jianhua Tang, Yao Wang, Pengyu Xu

**Affiliations:** 1 School of Intelligent Manufacturing, Changzhou Vocational Institute of Textile and Garment, Changzhou 213164, China; rbfan@cztgi.edu.cn (R.F.); gwshao@cztgi.edu.cn (G.S.); jdywang@cztgi.edu.cn (Y.W.); 2 The Jiangsu Research Center of Intelligent Manufacturing Technology for Carbon Fiber and Advanced Material, Changzhou 213164, China; 3 Engineering Research Center of Artificial Intelligence for Textile Industry Ministry of Education, Institute of Artificial Intelligence, Donghua University, Shanghai 201620, China; 4 School of Innovation and Entrepreneurship, Changzhou Vocational Institute of Textile and Garment, Changzhou 213164, China; 5 School of Material Science and Engineering, Zhengzhou University, Zhengzhou 450001, China

**Keywords:** industrial robots, adaptive grippers, compliant structures, active compliance, learning-based grasping, carbon fibre self-sensing grippers

## Abstract

The increasing demand for flexible, high-precision, and low-damage manufacturing is driving industrial robotic grippers toward adaptive systems with compliant interaction, force regulation, and intelligent decision-making capabilities. However, their industrial deployment remains limited by three major challenges: the trade-off between structural compliance and load-bearing capability, the insufficient coordination between sensing performance and closed-loop force control, and the limited generalization and safety validation of learning-based methods. This review presents an engineering-oriented analysis of adaptive robotic grippers based on three paradigms: structural compliance, active compliance, and learning-enabled grasping. The design principles, performance characteristics, and application limitations of compliant mechanisms, variable stiffness structures, carbon fiber composites, embedded sensing, force control, data-driven methods, reinforcement learning, and model–data fusion are discussed. A comparative framework is established according to the adaptation mechanisms, key parameters, performance boundaries, and industrial applications. The analysis shows that representative underactuated grippers typically achieve 3–19 degrees of freedom, grasping success rates of 90–98%, and load capacities of approximately 10–50 N, but remain limited in force regulation. Actively compliant grippers achieve force errors of 0.1–1 N, position errors of 0.05–0.5 mm, and control frequencies of 500 Hz–1 kHz, while learning-enabled methods require 10^2^–10^4^ samples for compensation and 10^5^–10^6^ interactions for reinforcement learning. The model–data fusion provides a practical balance between adaptability, interpretability, and safety. Future adaptive grippers will evolve toward integrated structure, sensing, learning, and control architectures with enhanced reliability and industrial applicability.

## 1. Introduction

Industrial robots are evolving from rigid automation systems designed for repetitive and standardized operations toward flexible manufacturing platforms capable of handling multi-variety and small-batch production. This transition requires robotic systems to achieve reliable manipulation under uncertain conditions involving irregular geometries, fragile surfaces, variable material properties, and dynamic contact interactions. Consequently, the adaptability and interaction capability of robotic end-effectors have become critical factors affecting manufacturing flexibility and operational reliability. As the component directly interacting with workpieces, robotic grippers play a key role in determining the grasping stability, production efficiency, positioning accuracy, and potential surface damage. Therefore, adaptive robotic grippers have emerged as an important research direction in intelligent manufacturing and flexible robotic systems (as shown in Figure 1).

Three complementary technological routes have progressively developed to improve gripper adaptability: structural compliance, active compliance, and learning-enabled grasping. Structural compliance introduces adaptability through mechanical deformation and has evolved from classical underactuated mechanisms toward soft and variable stiffness architectures. Birglen et al. [1] established systematic design and analysis principles for underactuated robotic hands, while Shintake et al. [2] reviewed the subsequent development of soft robotic grippers. Hughes et al. [3] further summarized the evolution of soft manipulators and grippers based on distributed material compliance. Active compliance instead introduces force, tactile, and pose sensing into closed-loop interaction control. Variable impedance strategies have become an important extension of this route because they allow the desired interaction dynamics to be adjusted according to changing task conditions [4]. More recently, learning-enabled manipulation has expanded from data-driven grasp synthesis toward reinforcement learning, multimodal perception, and generalist robot policies. Bohg et al. [5] systematically reviewed early data-driven grasp synthesis, whereas Kober et al. [6] established the broader foundations of reinforcement learning for robotic control.

Gripper selection is strongly constrained by the operational requirements of the target task. Conventional rigid grippers remain advantageous in highly structured production environments requiring high payload, high speed, and repeatable positioning, where the workpiece geometry and contact conditions are well defined. In contrast, structurally compliant grippers are more suitable for irregular, fragile, or geometry-variable objects because passive deformation can improve conformity and reduce local contact stress. For precision assembly and other contact-sensitive operations, active compliance becomes necessary when the grasping force and interaction stiffness must be regulated online. Learning-enabled approaches are more attractive in unstructured or multi-object scenarios where object properties, poses, and contact conditions vary substantially. Therefore, the payload capacity, manipulation speed, positioning accuracy, allowable contact force, and environmental uncertainty should be considered jointly when selecting an appropriate gripper architecture.

Against these different operational requirements, structural compliance, active compliance, and learning-enabled grasping should not be regarded as mutually exclusive device categories, but as complementary adaptation routes operating at different levels. Structural compliance embeds adaptability in the mechanical structure; active compliance regulates physical interaction through sensing and feedback control; and learning-enabled grasping extracts task-relevant representations or policies from the data and interaction experience. Their integration is increasingly important for industrial systems that simultaneously require low-damage contact, accurate force regulation, environmental adaptability, and verifiable safety.

Existing reviews have extensively addressed individual topics, such as soft robotics, tactile perception, impedance control, reinforcement learning, or intelligent grasping [7]. Polygerinos et al. [8], for example, systematically reviewed fluid-driven soft robotic systems from the perspectives of fabrication, sensing, and control. However, the engineering-oriented framework that connects mechanical compliance, embedded perception, force control performance, learning mechanisms, and industrial application requirements remains insufficient. This review therefore analyzes adaptive robotic grippers from the perspectives of structural compliance, active compliance, and learning-enabled manipulation, with particular attention to their adaptation mechanisms, representative performance ranges, technical limitations, application scenarios, and integration pathways (as shown in Table 1).

## 2. Structurally Compliant Grippers

Structurally compliant grippers introduce adaptability directly through their mechanical design and passive deformation. According to the primary compliance mechanism, the main structural routes can be broadly distinguished as underactuated mechanisms, soft structures, and variable stiffness structures. Underactuated mechanisms generate adaptive force redistribution through kinematic and transmission coupling; soft structures obtain compliance from continuous material deformation; and variable stiffness designs actively or passively alter structural stiffness to switch between conformability and load support. These routes are defined according to how mechanical compliance is generated and are therefore separated from enabling material technologies. Advanced materials, such as carbon fiber-reinforced composites, can be incorporated across different architectures to improve lightweight performance, directional stiffness, load-bearing capability, and sensing integration.

Compared with actively compliant grippers, structurally compliant designs generally require fewer sensors and lower control complexity and can rapidly adapt to uncertain geometries. Their principal limitations arise from the passive force distribution; limited online adjustability; and the inherent trade-off among compliance, load-bearing capability, positioning accuracy, and grasping stability.

### 2.1. Design of Underactuated Mechanisms

Underactuated mechanisms achieve coordinated multi-joint motion using fewer actuators than the available degrees of freedom. Their adaptive capability originates from the coupling among kinematic constraints, elastic elements, and force transmission paths. When one phalanx contacts the workpiece, the driving force can be redistributed to the remaining phalanges through linkages, tendons, or differential mechanisms, enabling multi-point or enveloping grasping without independent actuation of every joint. Underactuated grasping has developed from mechanically coupled multi-joint hands toward highly compliant architectures capable of robust passive adaptation. Birglen et al. [1] established the theoretical and design foundations of underactuated robotic hands and systematically analyzed linkage configuration, contact sequence, force transmission, and grasp stability. Dollar and Howe [9] subsequently demonstrated that compliant underactuation can achieve robust grasping in unstructured environments while maintaining low actuation and control complexity. Deimel and Brock [10] further combined underactuation with highly compliant finger structures, illustrating how mechanical intelligence can reduce sensing and low-level control requirements while retaining a dexterous manipulation capability. Underactuated grippers can generally be implemented using linkage-driven, tendon-driven, differential, or hybrid transmission mechanisms, as shown in Figure 2. Linkage-driven mechanisms achieve sequential closure through mechanical linkages, with the linkage length ratios and joint stiffness serving as major design parameters. Tendon-driven mechanisms use tendon pretension and elastic elements to couple multiple joints. Differential mechanisms distribute the driving forces among the fingers through gears or pulleys, whereas hybrid configurations combine multiple transmission principles to balance compactness, force distribution, and mechanical efficiency.

As summarized in Table 2, representative underactuated grippers exhibit approximately 3–19 DOFs, load capacities of 10–50 N, and grasping success rates of 90–98%. However, direct quantitative comparison remains difficult because object sets, manipulation tasks, loading conditions, and success criteria differ substantially among studies.

The main advantage of underactuated mechanisms is therefore their ability to achieve geometric adaptation with low sensing and control complexity. Their limitations include transmission losses, passive force distribution, dependence on joint stiffness, and limited online force regulation. Consequently, they are particularly suitable for irregular objects and uncertain poses when precise force tracking is not the dominant requirement.

### 2.2. Soft and Variable Stiffness Technologies

Soft grippers achieve conformal grasping through continuous deformation of compliant materials, such as silicone elastomers, thermoplastic polyurethane, and fiber-reinforced elastomers. Unlike underactuated mechanisms based primarily on discrete joint motion, soft grippers distribute the contact deformation over a continuous body, thereby reducing the local stress concentration and improving shape conformity. Lu et al. [14] emphasized this shift from rigid robotic body structures toward continuously deformable systems, in which the morphology directly contributes to the interaction capability. Li and Rus [15] subsequently summarized the fundamental design, fabrication, actuation, and control principles of soft robots. Shan et al. [16] further reviewed soft manipulators and grippers and highlighted their particular suitability for safe interaction and fragile object manipulation.

Existing soft gripper designs encompass pneumatic chambers, fiber-reinforced structures, fabric-based actuators, vacuum structures, and multi-segment soft fingers. Nguyen et al. [17] developed fabric-based soft grippers capable of selective distributed bending, illustrating how textile reinforcement and chamber configuration can regulate deformation. Wang and Hirai [18] demonstrated the practical use of soft robotic grippers for packaging deformable food products. Teeple et al. [19] developed multi-segment soft fingers capable of both precision and enveloping grasping. These studies demonstrate that the structural geometry and material distribution are as important as the intrinsic modulus of the elastomer (as shown in Figure 3).

Soft grippers are commonly fabricated using silicone, TPU, and fiber-reinforced elastomers, with pneumatic actuation being the dominant driving method. Shintake et al. [2] reviewed the materials, actuation methods, and structural designs of soft grippers, indicating that the material hardness, chamber geometry, and actuation pressure are the key factors affecting bending deformation and fingertip force. Manti et al. [20] developed an anthropomorphic three-finger soft gripper using pneumatic actuation and elastomeric materials, demonstrating the influence of finger thickness distribution and material modulus on bending behavior and output force. Tawk et al. [21] developed a fully 3D-printed vacuum-driven soft actuator and investigated the effects of chamber geometry, wall thickness, and material hardness on actuator deformation and force output. The material characteristics and corresponding grasping performance of representative soft grippers are compared as shown in Table 3.

Low-modulus elastomers provide large deformation and low contact stress but generally exhibit limited grasping force and positioning accuracy. Increasing the material modulus or introducing fiber reinforcement improves the load-bearing capability but reduces the free deformation. Therefore, material selection requires a balance among the deformation capability, contact compliance, structural support, and dynamic response.

Variable stiffness technologies provide an important solution to this trade-off by allowing a gripper to switch between compliant adaptation and higher stiffness support. Manti et al. [22] systematically reviewed stiffening mechanisms for soft robotics, including jamming, tendon-based stiffening, low-melting-point materials, and other phase-transition methods. Ilievski et al. [23] demonstrated large reversible deformation using pneumatically actuated elastomeric structures, establishing an important basis for subsequent soft robotic architectures (as shown in Table 4).

Granular jamming is one of the most widely investigated variable stiffness strategies. Brown et al. [24] demonstrated passive conformal grasping and shape locking based on particle jamming. Amend et al. [25] proposed a positive-pressure-driven granular jamming strategy, where particle rearrangement enabled adaptive contact and subsequent shape fixation. Yang et al. [26] combined granular jamming and fiber interference in a bioinspired soft robotic finger, achieving a substantial stiffness variation while retaining compliant deformation. Wang et al. [27] further integrated positive pressure and partial particle filling to improve the adaptability of particle-jammed grippers. Gao et al. [28] developed a multimode variable stiffness finger integrating granular jamming with antagonistic tendons, achieving a fivefold stiffness adjustment compared with the initial state. Zhou et al. [29] investigated nonlinear stiffness evolution of granular materials under different stress paths using DEM simulations, providing insights into mechanism modeling.

Soft compliance has also demonstrated advantages in demanding low-damage applications. Guo et al. [30] applied soft robotic grippers to biological sampling, showing that intrinsic compliance can enable stable manipulation of fragile specimens that are difficult to handle using conventional rigid mechanisms. This application-oriented evidence reinforces the role of soft structures when low contact damage is prioritized over high load or positioning stiffness.

Overall, soft structures provide high morphological adaptability and low contact stress, whereas variable stiffness mechanisms improve shape retention and load support after contact is established. Representative soft grippers provide grasping forces in approximately the 5–40 N range, while variable stiffness technologies exhibit response times ranging from tens of milliseconds for field-responsive materials to several seconds for thermally activated mechanisms. Their practical selection therefore depends on the required balance among compliance, response speed, payload, energy consumption, and stiffness retention.

### 2.3. Carbon Fiber Composites as an Enabling Technology for Compliant Grippers

Carbon fiber-reinforced composites do not constitute an independent compliance mechanism; rather, they serve as an enabling material platform that can be integrated into underactuated, compliant, and variable stiffness architectures.

Carbon fiber-reinforced composites should be regarded as an enabling structural technology rather than an independent compliance mechanism. Their high specific strength, low density, and tailorable anisotropic stiffness make them suitable for lightweight compliant fingers, reinforced soft structures, and high-speed gripper components. Kumpati et al. [31] employed a multi-objective genetic algorithm to optimize composite laminate stacking sequences, enabling directional stiffness design. Gao et al. [32] investigated the fatigue and fracture behavior of CFRP structures under cyclic loading, providing insights into the durability design of composite compliant components.

More importantly, recent studies have directly demonstrated the use of carbon fiber reinforcement in robotic fingers and grippers. Zhang et al. [33] developed an electrically actuated soft finger reinforced with a carbon fiber framework, achieving a more than threefold improvement in the output performance compared with a conventional polymeric structure. Jimbo et al. [34] developed a two-degree-of-freedom tendon-driven robotic finger using continuous-CFRP additive manufacturing, enabling integrated compliant deformation and grasping force transmission. Chen et al. [35] introduced a carbon fiber skeleton-reinforced photothermal actuator into a flexible gripper, which was capable of grasping objects approximately eight times its own weight. Hartomacıoğlu et al. [36] further applied carbon fiber-reinforced polyamide to an industrial robot gripper and reported a 71% reduction in weight together with a 12.4% increase in tensile strength.

Additive manufacturing has further expanded the structural design space of CFRP-enabled grippers. Zhang et al. [37] optimized short carbon fiber-reinforced polyamide for fused-filament fabrication, improving the mechanical performance of lightweight printed structures. Afanasev et al. [38] developed a six-axis robot-assisted continuous-fiber 3D printing process, providing a manufacturing route for geometrically complex CFRP components. These manufacturing strategies facilitate integrated fabrication of lightweight compliant structures, although the fiber placement accuracy, interlayer bonding, and production cost remain important limitations.

Embedded sensing provides another pathway for extending CFRP grippers from lightweight structures toward self-sensing systems. Gabardi et al. [39] embedded fiber Bragg grating sensors into carbon composite structures and demonstrated accurate strain measurement, providing a basis for integrating structural load bearing and deformation perception. For high-precision compliant systems, the hysteresis and creep associated with nonlinear structural behavior must also be considered; Chen et al. [40] developed a Maxwell-slip-based hysteresis model for nonlinear compliant actuators, providing a modeling basis for subsequent compensation strategies.

Overall, CFRP should not be considered a separate compliance mechanism, but rather a cross-cutting structural technology that can reinforce soft structures, enable lightweight compliant fingers, support industrial gripper optimization, and facilitate embedded sensing. Its main advantages arise from its high specific strength and tailorable anisotropic stiffness, while manufacturing consistency, structural prediction, sensing integration, and long-term hysteresis compensation remain important challenges for industrial deployment.

### 2.4. Summary

Structurally compliant grippers introduce adaptability through underactuated mechanisms, soft structures, and variable stiffness designs. Carbon fiber composites can be integrated across these architectures as an enabling material technology to improve lightweight performance, directional stiffness, and load-bearing capability. The common design objective of these approaches is to regulate the equivalent contact stiffness at the gripper–workpiece interface. Underactuated mechanisms achieve adaptive force distribution through joint stiffness and transmission mechanisms, while soft grippers reduce the contact stress through material deformation. Variable stiffness and carbon fiber composite structures further improve the balance between compliance, load-bearing capability, and directional stiffness (as shown in Figure 4).

## 3. Actively Compliant Grippers

Actively compliant grippers achieve programmable interaction regulation through sensory feedback and closed-loop control. Unlike structurally compliant grippers, which rely primarily on passive mechanical deformation, active compliance allows for the grasping force, end-effector pose, interaction stiffness, and damping to be adjusted online according to workpiece properties and task requirements. Its performance is determined jointly by contact perception, state estimation, controller design, sensing bandwidth, actuation capability, and coupled robot–environment stability.

### 3.1. Contact Perception and Force Feedback

Sensing systems provide the foundation for active compliance. Based on the sensing principle and installation configuration, representative approaches include wrist-mounted force/torque sensing, fingertip tactile sensing, embedded optical fiber sensing, proximity/vision sensing, and sensorless interaction force estimation (as shown in Figure 5).

Tactile perception has long been recognized as a key information source for robotic manipulation because it provides direct measurements of local contact force, pressure distribution, contact location, and slip state. Yousef et al. [41] systematically reviewed tactile sensing for dexterous in-hand manipulation and identified sensitivity, spatial resolution, bandwidth, and integration capability as major performance requirements. Kappassov et al. [42] further reviewed tactile sensing technologies for dexterous robotic hands and emphasized the importance of connecting the local contact perception with manipulation control.

Cirillo et al. [43] investigated tactile sensors for robotic parallel grippers, demonstrating the importance of local contact information for grasp-state characterization. Schindlbeck and Haddadin [44] integrated force feedback with passivity-based impedance control and demonstrated stable physical interaction during contact transitions. Cherubini et al. [45] further developed a multimodal interaction control framework combining multiple perception channels. These studies illustrate the progressive transition from isolated force measurement toward multimodal closed-loop physical interaction.

Wrist-mounted six-axis force/torque sensors are widely used in industrial robotic systems due to their high accuracy and broad bandwidth. They are suitable for assembly, polishing, and contact-transition tasks. Compared with wrist-mounted sensors, joint torque and fingertip tactile sensors provide information closer to the contact region and are more suitable for detecting local contact states, slip, and contact-area variations. Park et al. [46] developed an integrated fingertip tactile sensor based on an inverted frustum structure, achieving a measurement range of 30 N and a force resolution of 6 mN. Dong et al. [47] proposed a vision-enhanced proximity tactile sensor that combines distance perception and texture recognition, improving the transition between pre-grasp positioning and post-contact manipulation.

Sensorless force estimation reduces hardware requirements by estimating the contact forces from motor currents, joint states, or pneumatic pressures combined with dynamic models. Han et al. [48] proposed a high-order finite-time observer for industrial robots to estimate time-varying interaction forces, providing a solution for collision detection and impedance control. However, its estimation accuracy remains sensitive to model uncertainty, friction, transmission backlash, and structural deformation.

Fiber Bragg grating (FBG) sensors provide advantages, including compact size, electromagnetic interference resistance, and embedded integration capability, making them suitable for lightweight self-sensing grippers. Zhang et al. [49] demonstrated that wavelength-division and space-division multiplexing methods enable distributed strain sensing within a single robotic finger. Long et al. [50] developed an ultrathin triaxial FBG wrist force sensor with a sensitivity of 34.07 pm/N and a coupling error below 0.21%. Lai et al. [51] proposed an FBG array-based flexible force sensor with a sensitivity of 1169.04 pm/N and an RMSE of 0.12 N, enabling stable force feedback after integration into a soft gripper.

The sensing methods compared in Table 5 exhibit different advantages depending on the application requirements. Force/torque sensors provide high accuracy and bandwidth but require compensation for dynamic disturbances. Tactile sensors provide direct contact information but increase the integration complexity. FBG sensors are suitable for lightweight and embedded sensing applications due to their compact structure and electromagnetic immunity. Sensorless estimation reduces hardware requirements but remains dependent on model accuracy. Accordingly, the disturbance-resistance ratings in Table 5 are based on the dominant interference sources and the level of compensation required rather than on a universal error threshold across different sensing principles.

### 3.2. Active Compliance Control

Active compliance control regulates physical interactions by coordinating the contact force, end-effector pose, and motion trajectory. Its theoretical foundations originate from classical force control and impedance control frameworks. Yoshikawa et al. [52] proposed hybrid position–force control by separating constrained task directions into position-controlled and force-controlled subspaces. Hogan [53] subsequently introduced impedance control to regulate the desired dynamic relationship between manipulator motion and interaction force. Chiou et al. [54] reviewed the early development of robot force control and emphasized the importance of contact constraints, force sensing, and closed-loop stability.

Hybrid force–position control separates task-space constraints into force-controlled and position-controlled directions. It is suitable for assembly, insertion, and peg-in-hole tasks with predefined contact constraints. Li et al. [55] proposed a velocity loop-based hybrid force–position control method for robotic peg-in-hole assembly, improving the interaction stability through a spiral search strategy. Zou et al. [56] combined learning-based optimization with hybrid force–position control to improve the stability of hole-searching and insertion processes.

Impedance control establishes a desired mass–damping–stiffness relationship between the motion error and interaction force. Winiarski et al. [57] demonstrated Cartesian impedance control for redundant lightweight robots, showing its effectiveness for physical interaction. Ott et al. [58] further developed a unified formulation of impedance and admittance control. Variable impedance has subsequently become important for manipulation tasks in which the desired interaction characteristics change over time. Roveda et al. [59] and Roveda et al. [60] proposed a Q-learning-based predictive variable impedance control method, where the interaction dynamics were learned for online impedance adjustment.

The stability of active compliance cannot be inferred from the force-tracking accuracy alone. Meng et al. [61] analyzed stability conditions for variable impedance control and demonstrated that arbitrary online variation in impedance parameters cannot be assumed to preserve stable interaction. More recently, Simonic et al. [62] further unified force and impedance regulation within a common interaction control framework. Admittance control is particularly useful for position-controlled robotic architectures; Sharkawy et al. [63] reviewed variable admittance control methods in human–robot interactions and analyzed the influence of parameter tuning and safety mechanisms on system stability.

Adaptive and robust control methods have been introduced to compensate for uncertainties caused by friction, hysteresis, and variations in workpiece properties. Huynh et al. [64] combined iterative learning with fuzzy impedance control and optimized fuzzy rules through gradient descent, reducing grasping force errors during repeated operations (as shown in Table 6).

For the equivalent second-order impedance model, the damping ratio is defined as

$$\zeta =D/\left(2\sqrt{\mathrm{MK}}\right)$$

where *M*, *D*, and *K* denote the target inertia, damping, and stiffness, respectively. Figure 6 therefore presents the normalized step responses as functions of time for different fixed damping ratios, rather than responses plotted against the damping coefficient *D*.

Once contact is established, the interaction dynamics additionally depend on the mechanical properties of the environment. For a locally linear contact, the environment force can be represented as Fe = Keδ + Deδ, where Ke and De denote the environment stiffness and damping, respectively. The resulting closed-loop behavior is therefore determined jointly by the commanded impedance parameters and the environment dynamics. Schindlbeck et al. [44] demonstrated that passivity-based impedance regulation can improve stability during contact transitions. As the environment stiffness increases, the interaction generally becomes more sensitive to control delay, sampling effects, and inappropriate impedance tuning, thereby reducing the available stability margin. Abu-Dakka et al. [4] further highlighted the importance of variable impedance adaptation when interaction conditions change. Therefore, practical active compliance design should evaluate both the nominal impedance response and the stability of the coupled gripper–environment system over the expected range of contact stiffnesses.

Figure 6 illustrates the normalized step responses of an equivalent second-order impedance model under different damping ratios. Increasing the damping ratio from 0.2 to 0.7 reduces the response oscillation and decreases the overshoot from approximately 50% to below 5%. At ζ = 1, the system reaches critical damping without overshoot, whereas ζ > 1 produces an overdamped response with slower convergence. Therefore, appropriate damping selection is required to balance the response speed and oscillation suppression. These responses characterize the intrinsic free-space dynamics of the reference impedance model and do not represent the coupled dynamics after contact with an external environment.

### 3.3. Self-Sensing Structure Integration

CFRP structures provide a potential pathway for integrating lightweight load bearing with embedded sensing. FBG sensors can be incorporated during composite layup or structural manufacturing to measure the local strain while preserving compactness and electromagnetic immunity. Gabardi et al. [31] demonstrated embedded FBG strain sensing in carbon composite structures, providing an important foundation for structure–sensing integration.

CFRP laminates enable directional stiffness regulation through fiber orientation, stacking sequence, and layup configuration. This allows gripper fingers to maintain bending compliance in the grasping direction while providing sufficient stiffness in the tensile, torsional, and transverse directions. However, the viscoelastic behavior of resin matrices and sensor–matrix coupling can introduce hysteresis and creep, resulting in force estimation errors during long-term loading and repeated grasping. The quantitative cyclic calibration of FBG-CFRP structures has reported hysteresis errors of approximately 0.19–0.38% and repeatability errors of 0.24–0.89% [65]. In robotic gripping applications, an FBG-based force sensor exhibited a maximum hysteresis of 4.83% and an RMSE of 0.14 N during cyclic force measurements [51]. The Prandtl–Ishlinskii model and generalized Maxwell model are commonly used to characterize these nonlinear behaviors, while parameter identification and compensation strategies can further improve force control stability.

FBG sensors have become a widely adopted sensing solution for CFRP self-sensing structures due to their compact size, electromagnetic interference resistance, and multiplexing capability. Considering the anisotropic characteristics of fiber-reinforced composites, Zhou et al. [66] proposed a multidirectional strain-decoupling method based on FBG birefringence. This method enabled multidirectional strain measurement in quasi-isotropic laminates and improved the decoupling capability of complex strain states. Lu et al. [67] developed a dual-layer FBG sliding tactile sensor for robotic fingers. By optimizing the FBG embedding depth and array configuration within a PDMS elastomer matrix, the sensor achieved a sensitivity of 12.4 pm/N and an overall deviation below 5 pm. The sensor was capable of distinguishing between static contact and dynamic slip states, providing feedback for grasp-state recognition.

The characteristics of carbon fiber self-sensing grippers are compared as shown in Table 7. FBG sensing points located near load-transfer regions can improve the local force identification accuracy, while excessive sensing points increase system complexity and signal-decoupling requirements. CFRP self-sensing grippers provide advantages, such as lightweight construction, structural stiffness, and electromagnetic interference resistance, making them suitable for semiconductor handling, thin-walled component grasping, high-speed lightweight manipulation, and clean manufacturing environments. However, challenges remain in composite manufacturing consistency, embedded sensor durability, temperature compensation, and hysteresis modeling.

### 3.4. Summary

Active compliance overcomes the limited online adjustability of structurally compliant grippers through a closed-loop chain consisting of contact perception, state estimation, and force regulation. Its performance depends on accurate acquisition of contact force, slip state, and pose information, as well as stable force regulation under limited sensing and control bandwidth. Wrist-mounted force sensors provide high accuracy and a mature technology, while fingertip tactile sensors and FBG sensors offer closer measurement of local contact states. Sensorless force estimation reduces hardware requirements but remains sensitive to model uncertainty and system nonlinearities. Therefore, sensing selection should be determined by task accuracy, integration constraints, cost, and environmental conditions.

The performance of actively compliant grippers is also strongly influenced by the impedance parameters. Damping mainly determines contact force oscillation and impact attenuation, whereas stiffness controls the steady-state force response and position sensitivity. Appropriate impedance tuning is required to balance the response speed and grasping safety. Future development will focus on the integrated optimization of mechanical structures, sensing systems, and control strategies rather than independent improvement of individual components. CFRP self-sensing structures provide a promising approach for integrating lightweight structures, embedded perception, and closed-loop force control. However, hysteresis compensation, temperature drift, manufacturing consistency, and long-term reliability remain key challenges for industrial deployment (as shown in Figure 7).

## 4. Learning-Enabled Grasping: Learning Paradigms and System Integration

Active compliance control enables force–position regulation through force, tactile, and pose feedback, providing stable and interpretable manipulation in structured industrial scenarios. However, when uncertainties exist in workpiece geometry, stiffness, mass, surface friction, and initial pose, model-driven methods often require extensive parameter identification and controller tuning, increasing the cost of system adaptation. For soft grippers and carbon fiber composite grippers, structural deformation, material anisotropy, contact nonlinearity, hysteresis, and sensor drift further complicate accurate modeling. Therefore, purely model-based approaches face challenges in achieving high precision, safety, and generalization under complex manipulation conditions.

Learning-based grasping establishes mappings between sensory information, interaction experience, and control decisions through machine learning, deep learning, reinforcement learning, and physics-informed learning methods. These approaches reduce dependence on explicit models for complex contact processes and improve adaptability in uncertain and unstructured environments. Levine et al. [68] demonstrated the feasibility of end-to-end visuomotor control, where robotic actions were generated directly from image inputs. These studies indicate that learning-based methods are particularly suitable for nonlinear perception mapping and grasping policy optimization.

To avoid conflating learning paradigms with system-level architectures, learning-enabled grasping is organized here using a hierarchical framework. At the learning paradigm level, two principal routes are distinguished: data-driven learning and reinforcement learning. Data-driven learning establishes mappings from collected sensing and interaction data to grasping states or control variables, whereas reinforcement learning optimizes grasping policies through reward-guided interaction. At the system integration level, model–data fusion is treated as an architectural pattern in which learning modules are embedded within model-based controllers for estimation, compensation, or parameter adaptation. Embodied intelligence is considered a broader system-level framework characterized by multimodal perception, task understanding, and cross-task generalization rather than an independent learning paradigm. This hierarchical organization distinguishes how a model is learned from how learned components are integrated into a robotic grasping system.

As shown in Figure 8, the proposed framework separates learning paradigms from system-level integration. Data-driven learning and reinforcement learning represent two distinct mechanisms for acquiring learned models or policies, whereas model–data fusion specifies how these learned components are combined with conventional controllers. Embodied and VLA-based systems operate at a broader system level, integrating multimodal perception, learned policies, and task-level reasoning to improve cross-task generalization. These levels are complementary rather than mutually exclusive.

### 4.1. Data-Driven Learning for State Estimation and Compensation

In this review, data-driven learning refers to supervised or statistical learning from collected sensing and interaction data for state estimation, nonlinear mapping, and control compensation, distinguishing it from reward-driven reinforcement learning.

Data-driven robotic grasping has progressively shifted from task-specific grasp synthesis toward large-scale visuomotor learning. Zhang et al. [69] systematically reviewed early data-driven grasp synthesis and organized the existing methods according to object familiarity, representation, and grasp-transfer strategy. Pinto and Gupta [70] demonstrated the scalability of self-supervised physical grasp learning through 50,000 grasp attempts collected over approximately 700 robot hours. Levine et al. [71] further scaled real-robot data collection to learn closed-loop hand–eye coordination directly from visual observations.

Synthetic datasets and task-specific grasp representations have further improved data efficiency. Zhang et al. [72] developed Dex-Net 2.0 by combining synthetic point-cloud data with analytic grasp metrics. Christen et al. [73] introduced a generative grasp synthesis network for real-time closed-loop grasping. Fang et al. [74] subsequently established GraspNet-1Billion as a large-scale benchmark for general object grasping.

In industrial adaptive grippers, data-driven models are also widely applied to contact force estimation, slip recognition, hysteresis compensation, sensor decoupling, and stiffness estimation. Deisenroth et al. [75] demonstrated the advantages of Gaussian processes in small-sample learning and uncertainty estimation for safety-sensitive robotic control. Raissi et al. [76] proposed the PINN framework, which incorporates physical equations into the learning process to improve physical consistency. Li et al. [7] further emphasized the role of multimodal tactile feedback in slip detection and grasp stability regulation.

For functional comparison, representative data-driven approaches are discussed according to three dominant modeling roles: static mapping, temporal modeling, and physics-constrained estimation. These roles are not necessarily mutually exclusive; for example, temporal models can also incorporate physical constraints. Static mapping models establish direct relationships between sensing signals and grasping parameters, making them suitable for quasi-static grasping and calibration compensation. Temporal prediction models capture dynamic variations in contact force, slip, and hysteresis, which are suitable for dynamic manipulation with compliant grippers. Physics-constrained models incorporate contact mechanics, material properties, or boundary conditions into the learning process, improving model reliability under limited training data. The framework of data-driven grasping control is shown in Figure 9. Multisource sensing signals are first processed and then input into learning models for contact force estimation, slip detection, grasping force compensation, and impedance parameter adjustment. The inference time of these models is generally within the millisecond range, while the required training data volume depends on model complexity and task requirements.

Different learning models exhibit distinct application characteristics. MLPs provide fast inference with simple architectures and are suitable for low-dimensional signal mapping, such as force feedback, motor current, and joint position. CNNs are effective for extracting spatial features from images, tactile arrays, and FBG sensor distributions. LSTMs capture temporal dependencies and are suitable for dynamic contact modeling and slip prediction. Transformers provide strong capability for multimodal sequence modeling but require larger datasets and higher computational resources. GPR provides uncertainty estimation and is suitable for small-sample calibration and safety compensation. PINNs integrate physical constraints into neural networks and are suitable for systems with known structural properties, such as CFRP grippers with defined stiffness and sensing mechanisms, where the contact force and deformation/strain states can be estimated under structural and mechanical constraints. Diffusion policy-based models further improve multimodal action generation capability, although their real-time deployment remains limited by computational requirements and validation challenges [77].

Imitation and demonstration learning provide another important route for policy acquisition without explicit reward-driven exploration. Mandlekar et al. [78] investigated generalization across long-horizon manipulation tasks using human demonstrations. Mandlekar et al. [79] subsequently analyzed the factors affecting offline learning from demonstrations. Zhao et al. [80] introduced action chunking-based policy learning for fine-grained bimanual manipulation, illustrating the value of temporal abstraction for complex action sequences.

The characteristics of data-driven control methods are compared as shown in Table 8. Data-driven approaches provide advantages in nonlinear approximation, multisource sensing fusion, and compensation of complex contact behaviors. They are suitable for tasks such as force estimation, slip recognition, hysteresis compensation, and sensor drift correction. However, their performance remains limited by insufficient out-of-distribution generalization, uncertain safety boundaries, and dependence on training data coverage. Therefore, in industrial gripper systems, data-driven models are more suitable as auxiliary modules for state estimation and compensation rather than independent low-level safety controllers.

### 4.2. Reinforcement Learning

Reinforcement learning formulates grasping as an interaction process between an agent and the environment. The agent selects actions according to the current state, receives feedback through reward signals, and updates its policy to maximize cumulative rewards. Unlike supervised learning, reinforcement learning does not require predefined optimal actions and can acquire grasping strategies through interaction-based exploration. Therefore, it is suitable for grasp point selection, coordinated force–position control, and manipulation strategy optimization in unstructured environments.

Regarding robotic grasping, reinforcement learning has been extensively investigated for grasp point selection, continuous force–position regulation, motion optimization, and task-level manipulation. Quillen et al. [81] compared off-policy reinforcement learning methods for vision-based robotic grasping. Ibarz et al. [82] subsequently summarized practical lessons from deep reinforcement learning on physical robots, emphasizing data efficiency and safe training. Kalashnikov et al. [83] developed QT-Opt for large-scale vision-based robotic grasping, demonstrating the potential of high-volume interaction data for closed-loop grasp policy optimization.

Representative continuous-control algorithms include DDPG [84], SAC [85], TD3 [86], and PPO [87]. DDPG extends actor–critic learning to continuous actions, SAC improves exploration through entropy regularization, TD3 reduces value function overestimation, and PPO provides relatively stable policy updates. These algorithms have therefore become important baselines for robotic manipulation and force-related policy optimization.

The state–action–reward interaction process of reinforcement learning-based grasping is shown in Figure 10. State information obtained from vision, force, tactile, or other sensors is input into the policy network to generate grasping actions. The resulting actions modify the contact state, and the reward function evaluates task performance to update the policy. In robotic grasping, reward design should incorporate not only grasping success but also force regulation, slip avoidance, motion smoothness, and safety constraints.

Figure 10 illustrates the state–action–reward closed loop in reinforcement learning-based grasping. State information is first fed into the policy network to generate actions. These actions are applied to the environment and change the contact state. The reward function then evaluates the execution outcome according to the task objective and drives policy updates. The key to this closed loop is not merely increasing the cumulative reward, but transforming engineering objectives into a learnable and safety-constrained reward structure.

The characteristics of reinforcement learning algorithms are compared as shown in Table 9. DQN is mainly applied to discrete decision problems, such as grasp point selection, whereas DDPG and TD3 are suitable for continuous control tasks. SAC provides strong exploration capability for complex contact optimization, while PPO is widely used as a baseline algorithm due to its stable training process. QT-Opt demonstrates the potential of large-scale visual grasping based on closed-loop interaction.

Direct trial-and-error learning on physical robots presents substantial risks, including collision, workpiece damage, gripper wear, and overload. Simulation-to-real transfer is therefore widely adopted. Zhao et al. [88] reviewed Sim-to-Real transfer in deep robotic reinforcement learning. Tobin et al. [89] introduced domain randomization for visual transfer, whereas Peng et al. [90] randomized the physical dynamics parameters to improve control policy robustness.

Safety during learning and deployment remains a central obstacle to industrial application. García and Manguri [91] systematically reviewed safe reinforcement learning and distinguished approaches based on modified objectives and constrained exploration. Phillips et al. [92] further linked safe reinforcement learning with learning-based robotic control and emphasized uncertainty estimation, formal constraints, and backup controllers. For adaptive grippers, these safeguards are especially important because policy errors can immediately result in overload, slip, collision, or surface damage.

The simulation-to-real transfer framework for reinforcement learning-based grasping is shown in Figure 11. Policies are first trained in simulation or digital-twin environments and then adapted using limited real-world data. Safety-monitoring mechanisms are introduced to restrict overload, slip, and collision risks. Simulation training typically involves 10^5^–10^6^ interaction samples, while real-world fine-tuning can be reduced to approximately 10^2^–10^3^ samples.

Embodied intelligence is not treated here as a separate learning paradigm. Instead, VLA-based embodied systems are regarded as a system-level extension that can incorporate data-driven and reinforcement learning components within multimodal perception–action architectures. Ramadan et al. [93] developed RT-1, demonstrating transformer-based policy learning from large-scale real-world robot data. Walke et al. [94] released BridgeData V2 to support scalable multi-task manipulation learning. The Open X-Embodiment initiative [95] subsequently aggregated heterogeneous datasets across multiple robot embodiments and demonstrated the potential of cross-robot learning. Khazatsky et al. [96] further expanded real-world manipulation diversity through the DROID dataset.

Generalist policies have increasingly targeted cross-task transfer. Khansari et al. [97] demonstrated zero-shot task generalization through language-conditioned imitation learning. Ghosh et al. [98] developed Octo as an open-source generalist robot policy trained on heterogeneous manipulation datasets. Brohan et al. [99] proposed RT-2, which transfers semantic knowledge from vision–language models to robotic action generation. Ishitoya et al. [100] subsequently introduced OpenVLA as an open vision–language–action model for robot control. Mandischer et al. [101] reviewed the rapid development of VLA models and highlighted data scaling, embodiment transfer, physical grounding, inference efficiency, and safety validation as major barriers to real-world deployment.

These systems differ from conventional task-specific learning primarily in model scale, multimodal conditioning, task generality, and cross-embodiment transfer rather than constituting a fundamentally separate learning paradigm.

### 4.3. Model–Data Fusion

Model–data fusion is not treated as an independent learning paradigm, but as an integration architecture that combines model-based control with learned estimation or compensation modules. Purely data-driven methods provide strong nonlinear approximation capability, but their sample efficiency, interpretability, and stability are often insufficient for high-reliability industrial grasping. In contrast, model-driven methods provide clear physical meaning and verifiable stability, but they are less effective at handling complex contact behavior, compliant deformation, and unknown operating conditions. Model–data fusion combines these two paradigms by using physical models and classical controllers to provide safety boundaries, while learning models compensate for residual errors and unknown nonlinearities.

Several studies have shown the advantages of model-assisted learning. Chua et al. [102] proposed a probabilistic dynamics-based reinforcement learning method that improved sample efficiency. Janner et al. [103] analyzed the effect of model prediction errors on policy optimization and emphasized the need for uncertainty constraints in learned models. Nagabandi et al. [104] combined neural network dynamics models with model-free reinforcement learning and improved learning efficiency through simulation pretraining and real-world fine-tuning. Johannink et al. [105] demonstrated residual reinforcement learning, in which a learned residual policy augments a conventional controller.

In gripper control, model–data fusion usually adopts impedance control, hybrid force–position control, or model predictive control as the baseline framework. The learning module is then used to estimate quantities that are difficult to model accurately, such as friction, hysteresis, sensor drift, compliant deformation, and contact stiffness variation. A typical fusion architecture consists of a model-based controller, a learning-based compensation module, and a safety-monitoring module. Task commands, the target gripping force, and pose information are first processed by the model-based controller to generate the baseline control output. The learning module then estimates model residuals from the sensing feedback and outputs compensation terms. The safety module constrains the compensation magnitude through anomaly detection and uncertainty evaluation, thereby preserving stability and safety under abnormal conditions (as shown in Figure 12).

Representative model–data fusion mechanisms include residual learning, adaptive parameter learning, physics-informed learning, and learning-based model predictive control. These mechanisms can be combined within the same architecture depending on the control objective and available physical knowledge. Residual learning uses neural networks or Gaussian processes to estimate the difference between model predictions and real observations and then applies the estimated residuals as compensation. Adaptive parameter learning estimates environmental stiffness, contact damping, or workpiece compliance online for controller adjustment. Physics-informed learning incorporates contact mechanics, structural stiffness, and material constraints into model training to improve physical consistency. Learning-based model predictive control uses learned system models to predict future states and generates control inputs through receding-horizon optimization.

Model–data fusion provides a better balance among interpretability, data demand, safety, and environmental adaptability than purely model-driven or purely data-driven methods. For industrial grasping, this balance is more important than the laboratory success rate alone because long-term stability, anomaly protection, maintainability, and verifiable safety boundaries are also required (as shown in Table 10).

Uncertainty evaluation is a key safety mechanism in fusion control. Since learning modules may produce unreliable outputs outside the training distribution, uncertainty-aware methods, such as Gaussian processes, Bayesian neural networks, ensemble learning, and confidence-gating mechanisms are often introduced. When predictive uncertainty exceeds a preset threshold or, equivalently, when the normalized prediction confidence falls below a confidence threshold, the controller should reduce the weight of learning-based compensation and switch to a conservative model-driven mode. In representative robotic applications, confidence thresholds ranging from approximately 0.5 to 0.9 have been adopted according to task-specific precision–recall trade-offs or experimentally validated success rates [106]. Therefore, the threshold should be calibrated using validation data and task-specific safety requirements rather than treated as a universal constant. This safety fallback strategy retains the benefit of learning-based compensation while reducing the risk of overload, slip, and collision caused by model failure (as shown in Figure 13).

### 4.4. Learning for Carbon Fiber Self-Sensing Grippers

Carbon fiber composites exhibit high specific strength, high specific stiffness, low density, and excellent fatigue resistance, making them suitable as lightweight grippers and high-speed end-effectors. However, their mechanical behavior is strongly influenced by their fiber orientation, layup configuration, resin properties, interfacial bonding, and manufacturing defects, resulting in anisotropic deformation and structural coupling. In carbon fiber grippers, bending, torsion, contact deformation, and hysteresis further increase the difficulty of accurate contact force estimation. Polygerinos et al. [8] reported that soft robotic end-effectors provide high adaptability but remain difficult to model accurately due to their nonlinear deformation characteristics. Ding et al. [107] further demonstrated that sensing-domain discrepancies in tactile Sim-to-Real transfer can affect the accuracy of real-world robotic grasping.

Carbon fiber self-sensing grippers integrate sensing elements into composite structures to achieve simultaneous load bearing, deformation measurement, and state perception. Existing sensing approaches include FBG sensing, piezoresistive sensing, capacitive sensing, conductive carbon fiber sensing, and flexible tactile arrays. Among these methods, FBG sensing is widely adopted for CFRP-based self-sensing structures due to its compact size, electromagnetic interference resistance, multiplexing capability, and compatibility with composite manufacturing processes.

FBG sensors estimate local strain states through wavelength shifts caused by mechanical deformation and temperature variations. In carbon fiber grippers, FBG sensors are typically embedded near the finger root, major bending regions, and contact-sensitive areas to monitor deformation, contact force variation, and slip behavior. However, the relationship between FBG signals and contact force is affected by temperature drift, material anisotropy, and multi-sensor coupling. Therefore, conventional single-point calibration methods are insufficient for force estimation under complex operating conditions. Lu et al. [108] demonstrated the capability of FBG-based tactile sensing for dynamic contact-state recognition in robotic fingers.

Learning-based methods provide a pathway for nonlinear decoupling and force control compensation in FBG-based self-sensing grippers. By collecting multi-channel FBG wavelength-shift signals, gripper displacement, actual contact forces, and workpiece-state data, CNN, LSTM, GPR, or PINN models can be trained to establish mappings from FBG signals to the contact force, bending morphology, and slip state. Figure 14 illustrates the learning-based force control mechanism of carbon fiber self-sensing grippers. An embedded FBG network acquires multi-channel wavelength-shift signals, which are fed into the learning model after temperature compensation and preprocessing. The model outputs the contact force, bending state, and slip tendency. The fusion controller then adjusts the gripping force accordingly, while the safety module performs anomaly detection, overload protection, and control fallback.

The key issues and optimization strategies of FBG-based self-sensing grippers are compared as shown in Table 11. Temperature drift mainly affects signal stability and requires compensation methods based on reference sensing or temperature models. Multi-channel coupling caused by composite anisotropy requires nonlinear signal decoupling methods. Hysteresis and creep effects can be compensated for using sequence-learning models or residual correction strategies. Limited training data remains a challenge for model generalization, which can be improved through transfer learning and simulation data augmentation. In addition, confidence evaluation and fallback control are required to ensure safe operation when learning models produce uncertain outputs.

Representative learning models for carbon fiber self-sensing grippers are compared as shown in Table 12. Static mapping models provide rapid force estimation but are limited in their dynamic contact characterization. CNNs are suitable for extracting spatial features from FBG arrays, whereas LSTMs are more effective for modeling temporal behaviors, such as slip and hysteresis. GPR provides uncertainty estimation for small-sample calibration but becomes computationally expensive with increasing data size. PINNs improve physical consistency by incorporating structural and contact mechanics constraints, although accurate constraint formulation remains challenging.

From an engineering perspective, carbon fiber self-sensing grippers require integrated design of structure, sensing, learning, and control. FBG sensor placement should be determined according to the load-transfer paths and major deformation regions. Learning models should consider variations in workpiece stiffness, grasping position, temperature, and loading conditions to improve generalization. During control implementation, learning outputs should be used for force estimation and compensation rather than replacing low-level safety controllers. In practical deployment, anomaly detection and safety fallback strategies are required to handle sensor failures, model uncertainty, and abnormal contact conditions.

### 4.5. Summary

The primary role of learning-based grasping is to compensate for complex contact behaviors, sensing nonlinearities, and uncertain operating conditions rather than replace conventional control methods. At the learning paradigm level, data-driven learning is primarily used for force estimation, slip detection, state mapping, and nonlinear compensation, whereas reinforcement learning focuses on interaction-based policy and grasp strategy optimization. These learning paradigms can subsequently be embedded within model–data fusion architectures, where model-based controllers provide physical constraints and low-level stability while learned modules compensate for modeling errors and unknown nonlinearities. Embodied and VLA-based systems operate at a broader system level and extend these learning components toward multimodal task understanding and cross-task generalization rather than constituting an independent learning paradigm. The literature evolution reflects this hierarchy. Fatunmbi et al. [109] established an early taxonomy of data-driven grasp synthesis, while subsequent studies expanded toward large-scale real-robot learning. Brunke et al. [104] emphasized the increasing importance of safety mechanisms in learning-based robotic control. More recently, Kawaharazuka et al. [106] summarized the development of VLA systems toward multimodal and cross-task robotic manipulation ((as shown in Figure 15).

For carbon fiber self-sensing grippers, FBG-based distributed sensing provides internal structural information, the learning models extract nonlinear features from multisource signals, and model-driven controllers maintain safety constraints. Future research should focus on temperature compensation, force decoupling, physics-informed learning, digital-twin-based data augmentation, safety fallback strategies, and long-term reliability validation. The future development of intelligent grasping should emphasize the integration of lightweight structures, embedded sensing, physics-informed learning, and safe force control.

## 5. Conclusions and Outlook

### 5.1. Conclusions

This review summarizes recent advances in adaptive robotic grippers from the perspectives of structural compliance, active compliance, and learning-enabled grasping. The main conclusions are summarized as follows:

(1) Structurally compliant grippers achieve passive adaptation through mechanical architectures, including underactuated mechanisms, soft materials, and variable stiffness structures. Underactuated designs can realize multi-degree-of-freedom grasping using a limited number of actuators, with representative systems achieving approximately 3–19 DOFs, grasping success rates of 90–98%, and load capacities of 10–50 N. Their advantages include their simple structures, rapid response, and low control complexity. However, the lack of active force regulation and limited online stiffness adjustment restrict their application in precision assembly and high-consistency manufacturing scenarios.

(2) Actively compliant grippers improve the interaction capability through multisource sensing, force feedback, and impedance-based control strategies. Representative systems achieve force control errors of 0.1–1 N, positioning errors of 0.05–0.5 mm, and control frequencies of 500 Hz–1 kHz. Among the impedance parameters, damping mainly affects transient vibration suppression and contact stability, while stiffness determines the steady-state force response and positioning sensitivity. Despite improved controllability, active compliance remains dependent on sensing accuracy, model consistency, and system calibration, resulting in increased hardware and control complexity.

(3) Learning-enabled grasping extends adaptive manipulation toward uncertain and unstructured environments through data-driven control, reinforcement learning, and model–data fusion. Data-driven methods typically require 10^2^–10^4^ samples for force estimation, slip recognition, and nonlinear compensation, whereas reinforcement learning generally requires 10^5^–10^6^ simulation interactions for policy optimization. Model–data fusion provides a practical balance among adaptability, interpretability, and safety by combining learning-based compensation with model-driven control. However, insufficient out-of-distribution generalization, data efficiency, and safety validation remain key challenges for industrial deployment.

(4) Carbon fiber self-sensing structures provide a promising pathway for integrating lightweight mechanical design, embedded sensing, and intelligent force regulation. By combining CFRP structures with FBG sensing and learning-based compensation, adaptive grippers can simultaneously achieve load-bearing capability, deformation monitoring, and contact-state recognition. However, challenges remain in temperature compensation, hysteresis modeling, sensor integration reliability, and long-term operational stability.

### 5.2. Outlook

Future adaptive robotic grippers are expected to evolve from independent optimization of structures, sensing systems, and control algorithms toward integrated structure, sensing, learning, and control architectures (as shown in Figure 16). From an application-oriented perspective, gripper selection should be matched to the task requirements rather than treated as a universal design choice. Structurally compliant grippers are preferable for irregular, fragile, and geometry-variable objects where low contact damage, rapid passive adaptation, and low system complexity are prioritized. Actively compliant grippers are more suitable for precision assembly, semiconductor handling, and contact-sensitive manipulation requiring accurate force regulation and stable interaction. Learning-enabled grasping is better suited to unstructured or multi-object environments with substantial variations in object pose, stiffness, and contact conditions, while model–data fusion is particularly attractive for industrial tasks that require both adaptability and verifiable low-level safety.

First, lightweight structures with high stiffness and adaptive compliance will become an important development direction. The combination of carbon fiber-reinforced composites, variable stiffness mechanisms, and advanced manufacturing technologies is expected to further improve the balance between load-bearing capability, deformation adaptability, and dynamic response. Second, embedded sensing technologies will promote the transition from external perception toward intrinsic state awareness. FBG-based sensing, flexible tactile arrays, and multimodal sensing systems will provide more direct information on deformation, contact force, and slip behavior. Future research should focus on sensor calibration, temperature compensation, and robust signal decoupling for practical deployment. Third, physics-informed learning and uncertainty-aware control will become essential for improving the reliability of intelligent grasping. Learning models should be combined with physical constraints, digital twins, and safety fallback strategies to enhance the generalization capability while maintaining stable low-level force control. At the system level, future research should focus on three major challenges: rapid force estimation under complex contact conditions, cross-workpiece and cross-scenario generalization, and long-term reliability validation. Digital-twin-driven optimization and virtual–real collaborative learning are expected to provide effective approaches for accelerating the transition of adaptive grippers from laboratory prototypes to industrial applications.

However, each route retains distinct technical limitations. Structurally compliant grippers remain constrained by the trade-off between compliance and load-bearing capability, as well as limited online force adjustability. Actively compliant systems require high-quality sensing, sufficient control bandwidth, accurate calibration, and robust interaction stability, which increase the hardware and integration complexity. Learning-enabled approaches still face challenges in data efficiency, out-of-distribution generalization, interpretability, and safety validation. For industrial deployment, these limitations suggest that future development should emphasize hybrid architectures that combine mechanical adaptability, reliable sensing, model-based safety constraints, and learning-based compensation rather than relying on a single adaptation mechanism.

## Figures and Tables

**Figure 1:**
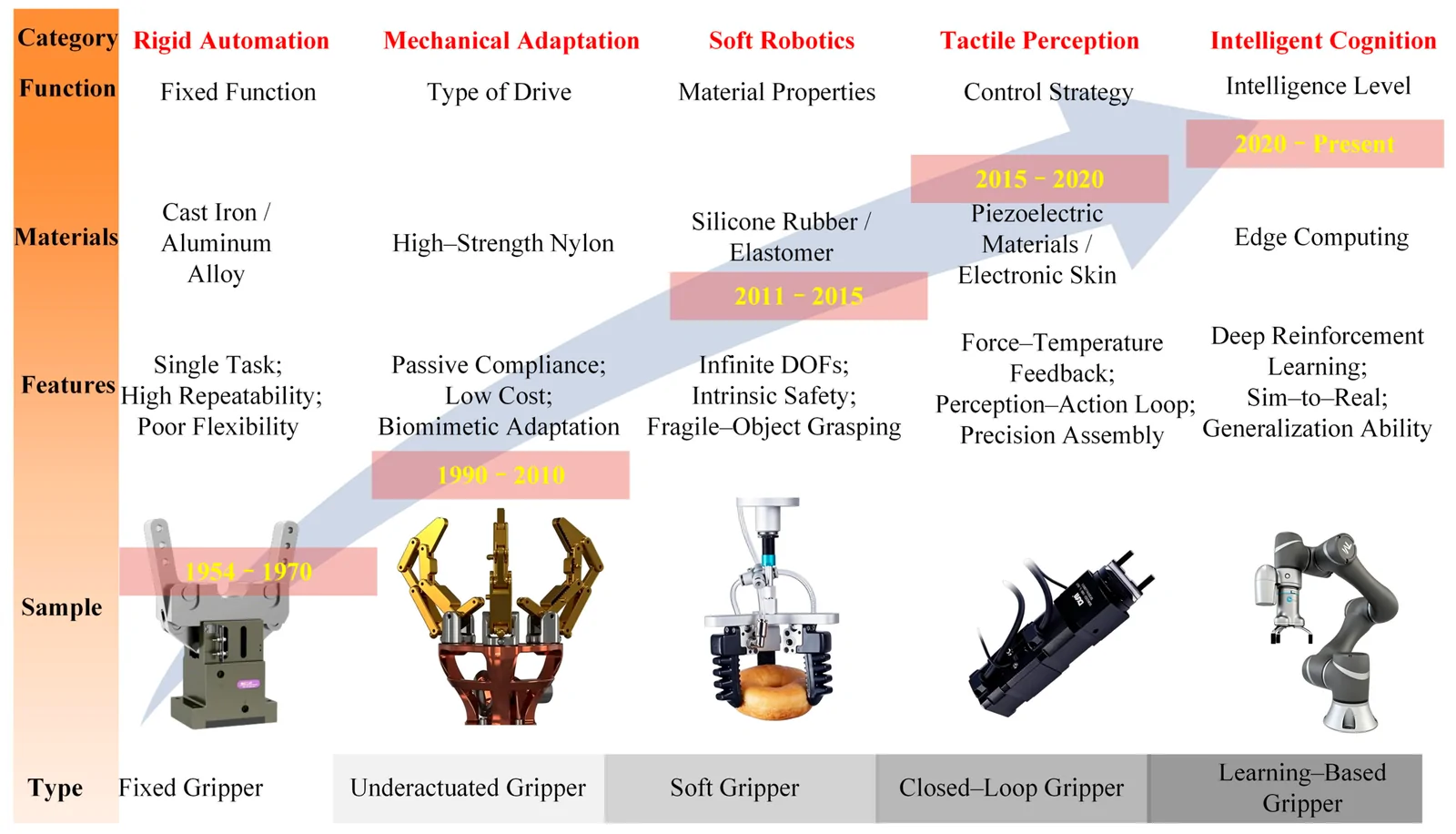
Evolutionary development of industrial robotic grippers from rigid mechanisms to intelligent adaptive systems.

**Figure 2:**
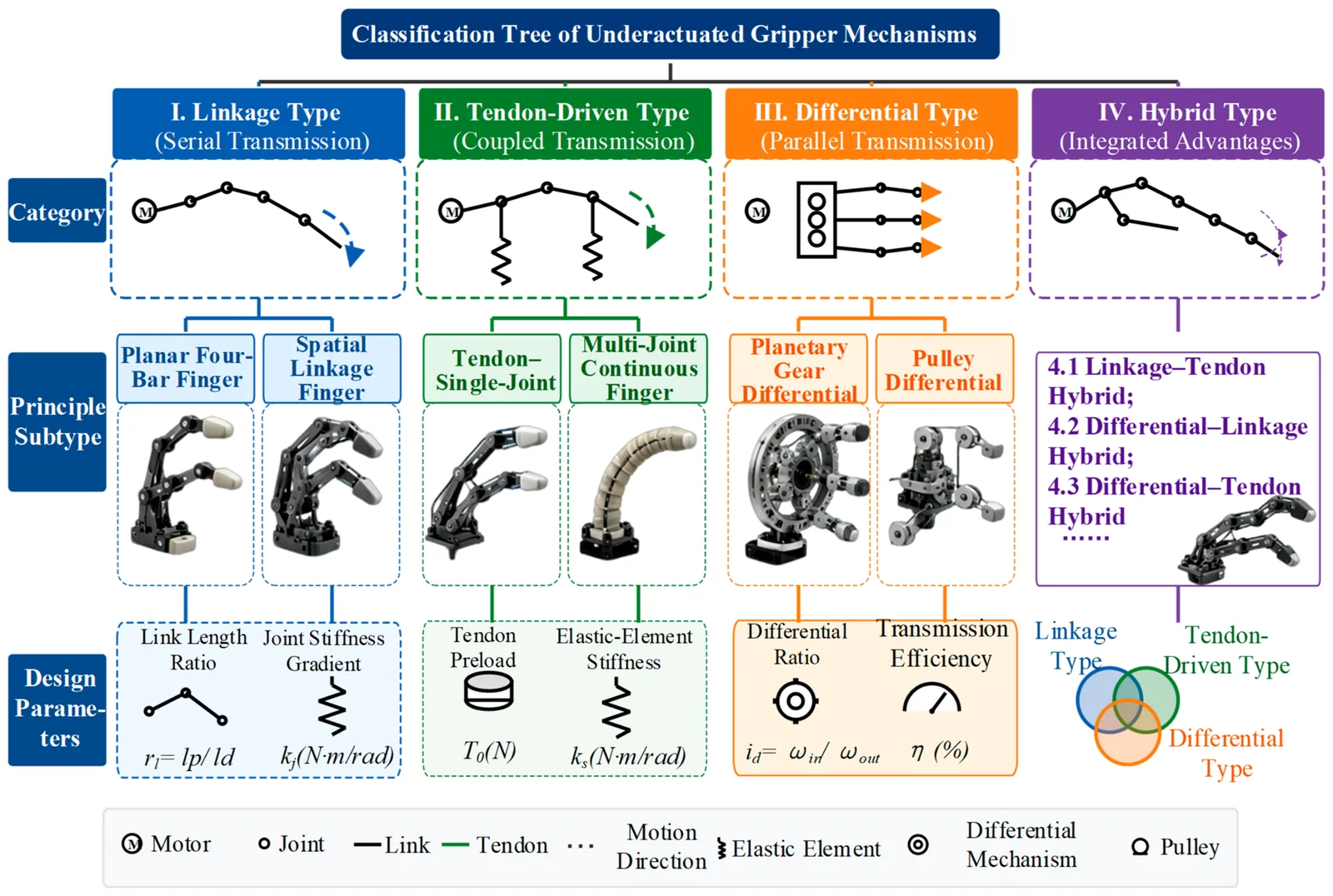
Classification of underactuated gripper mechanisms.

**Figure 3:**
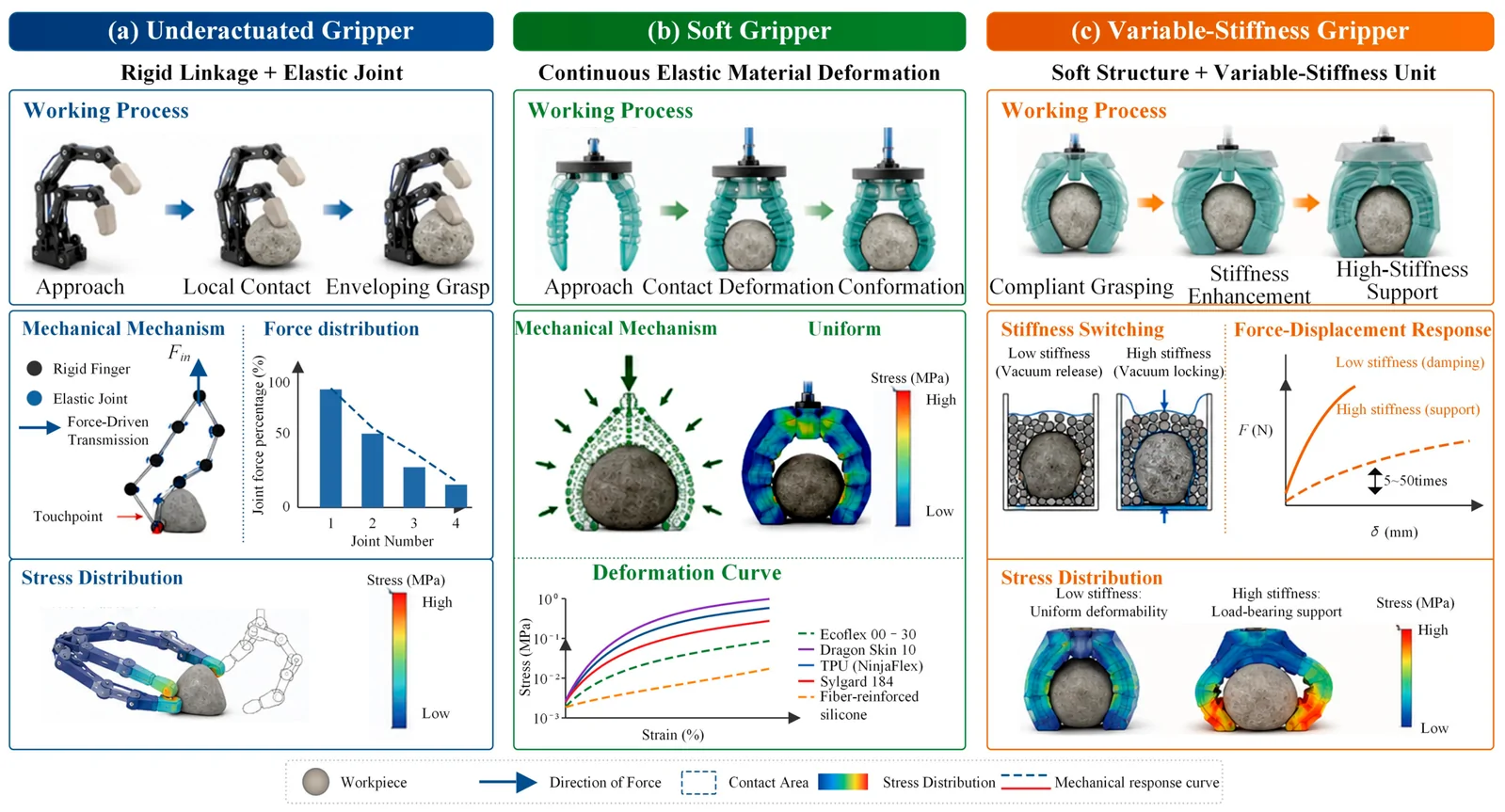
Comparison of structurally compliant gripper configurations.

**Figure 4:**
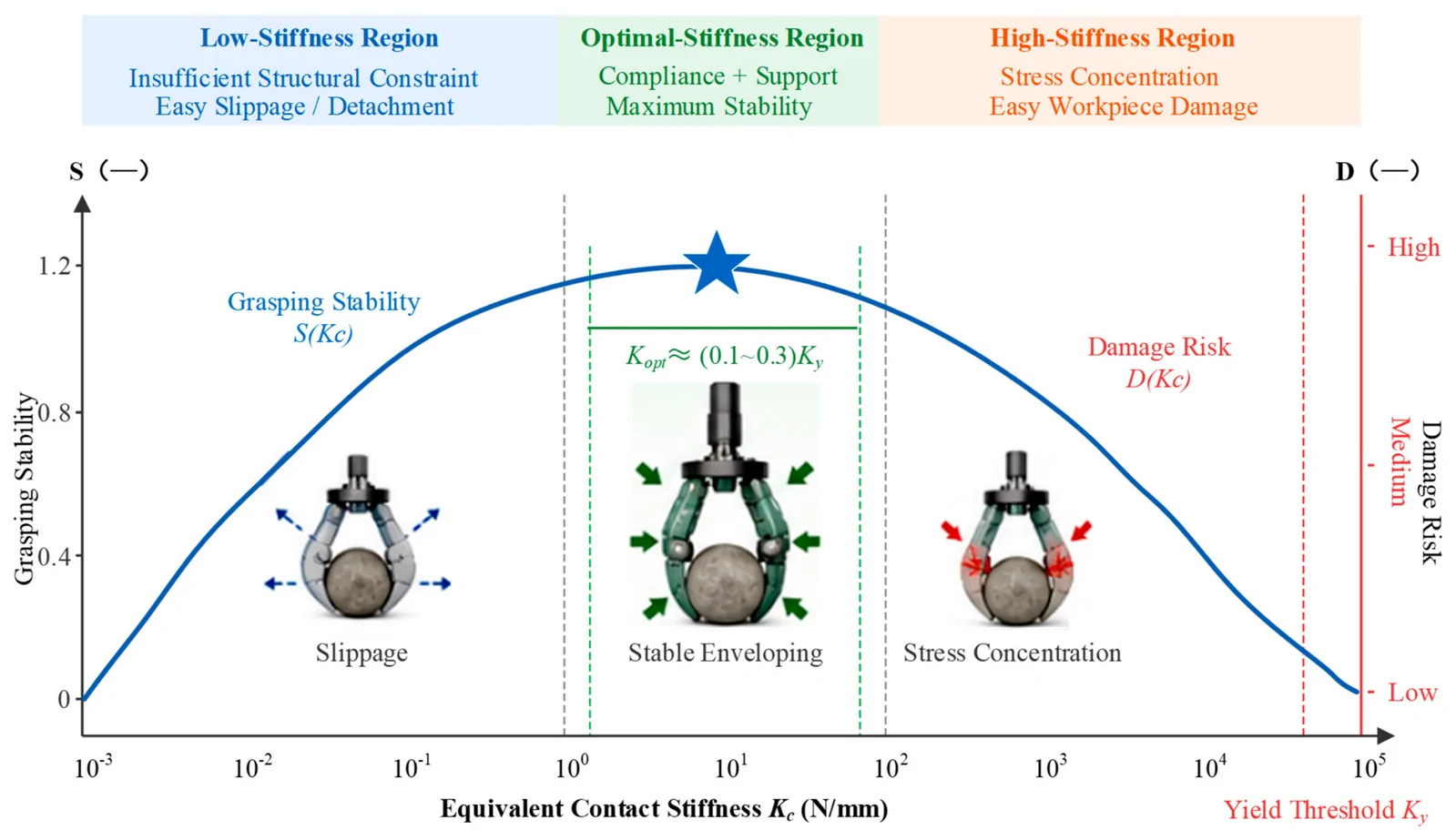
Relationship between contact stiffness and grasping stability.

**Figure 5:**
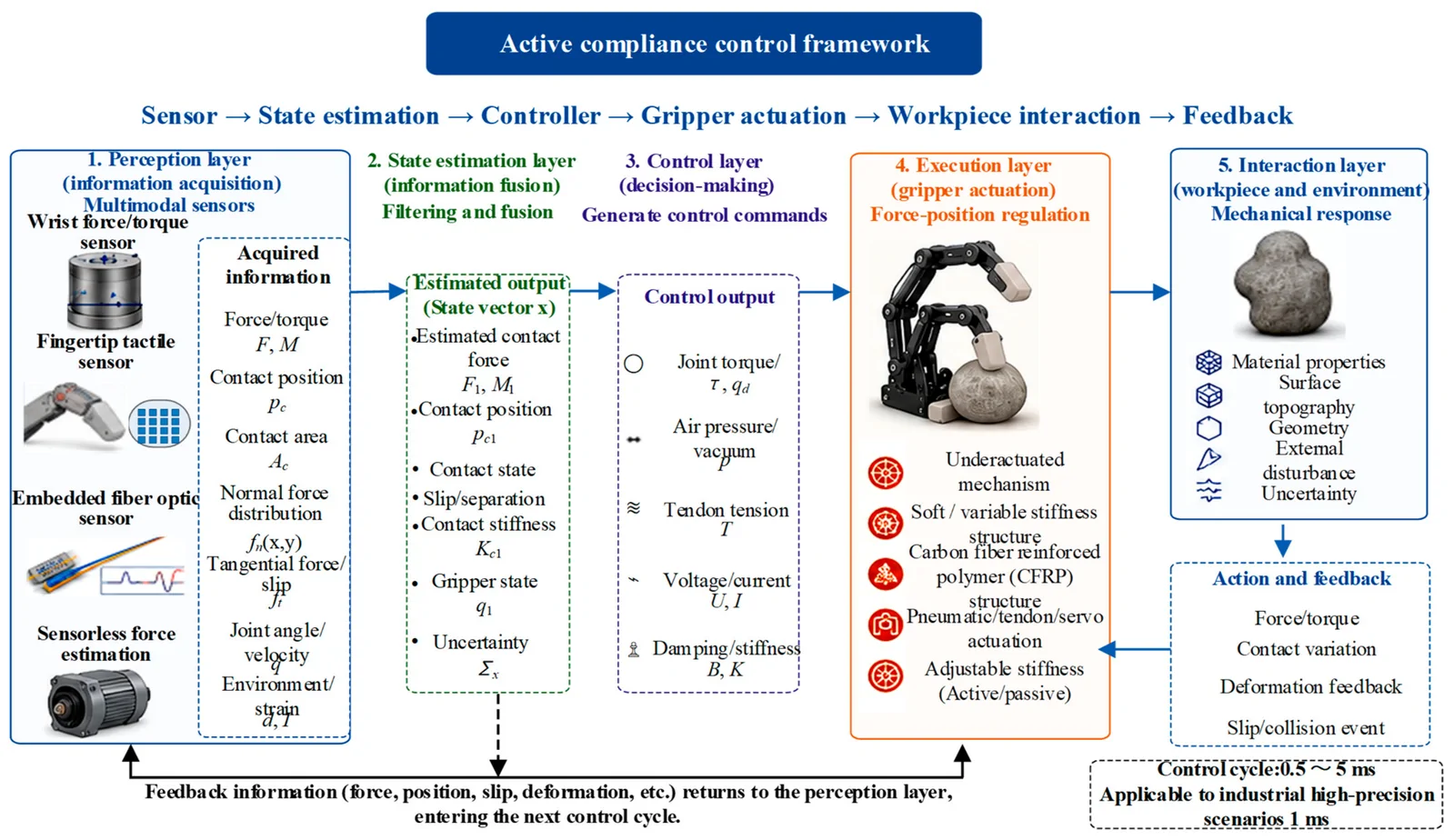
Closed-loop architecture of active compliance control.

**Figure 6:**
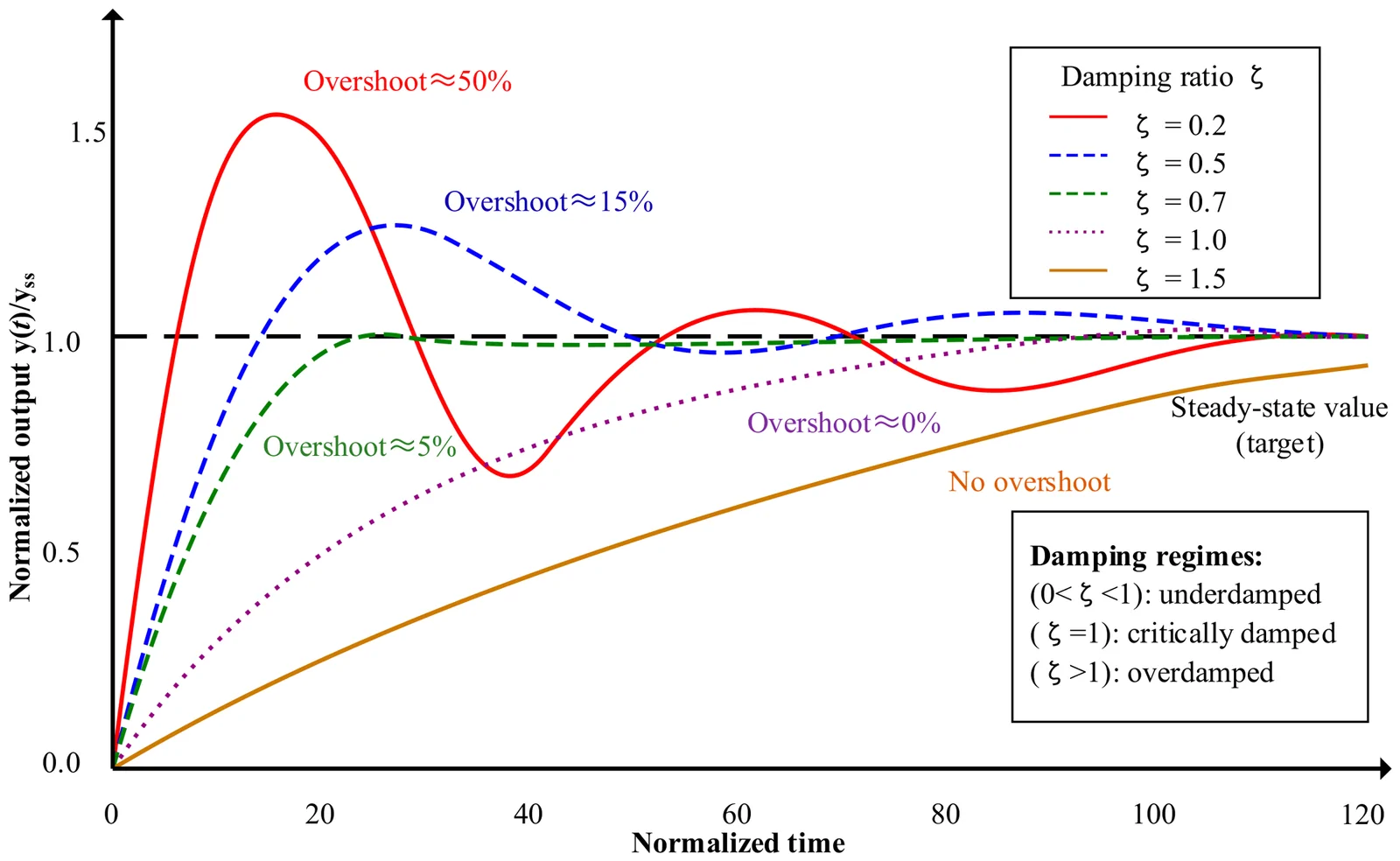
Normalized free-space step responses of the reference impedance model under different damping ratios.

**Figure 7:**
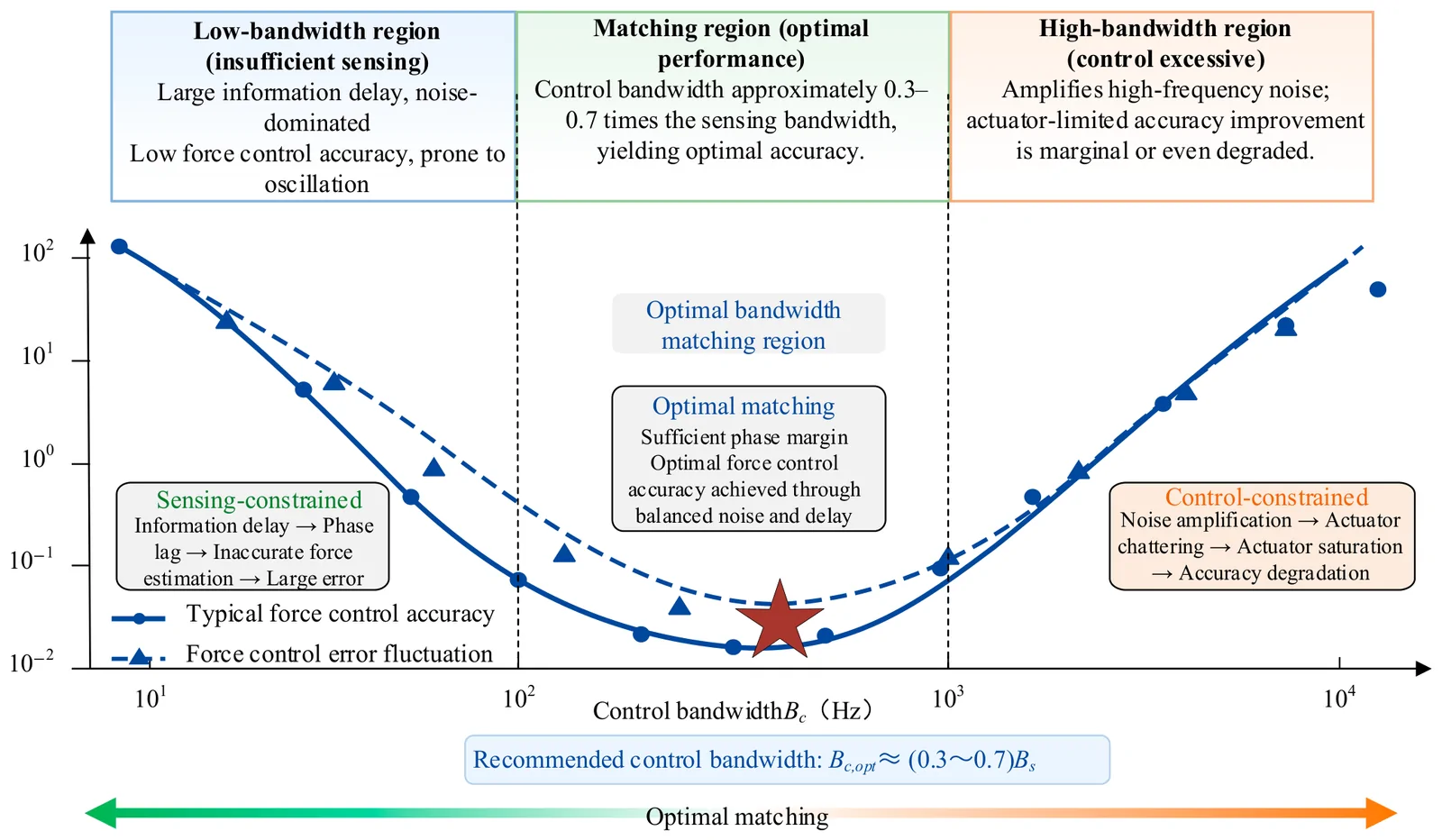
Relationship among sensing bandwidth, control bandwidth, and force control accuracy.

**Figure 8:**
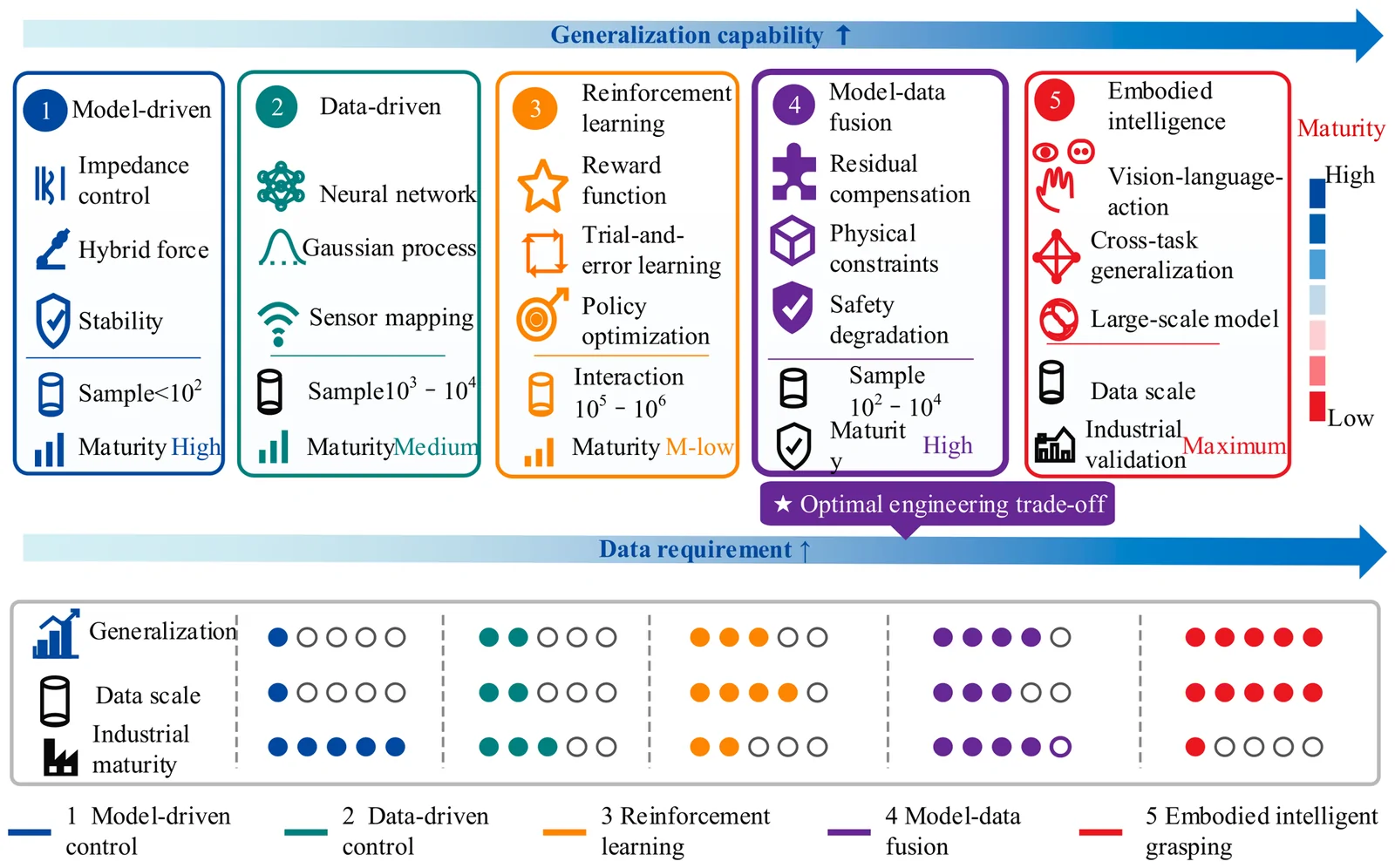
Development roadmap of learning-based grasping strategies.

**Figure 9:**
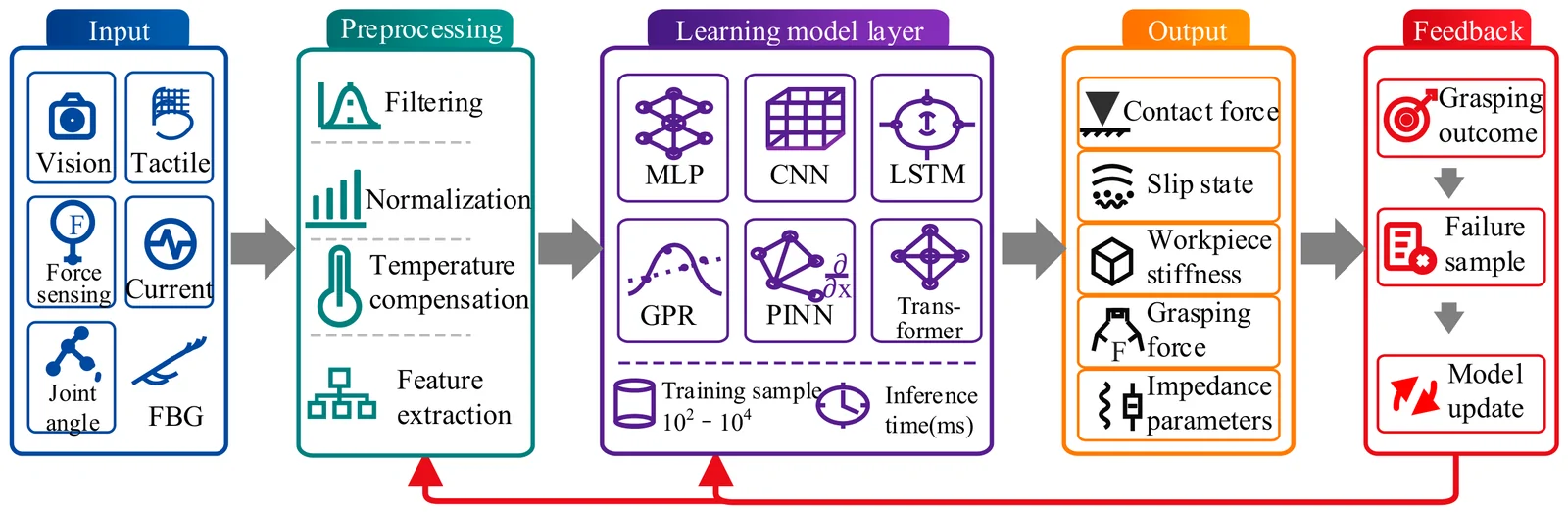
Framework of data-driven grasping control.

**Figure 10:**
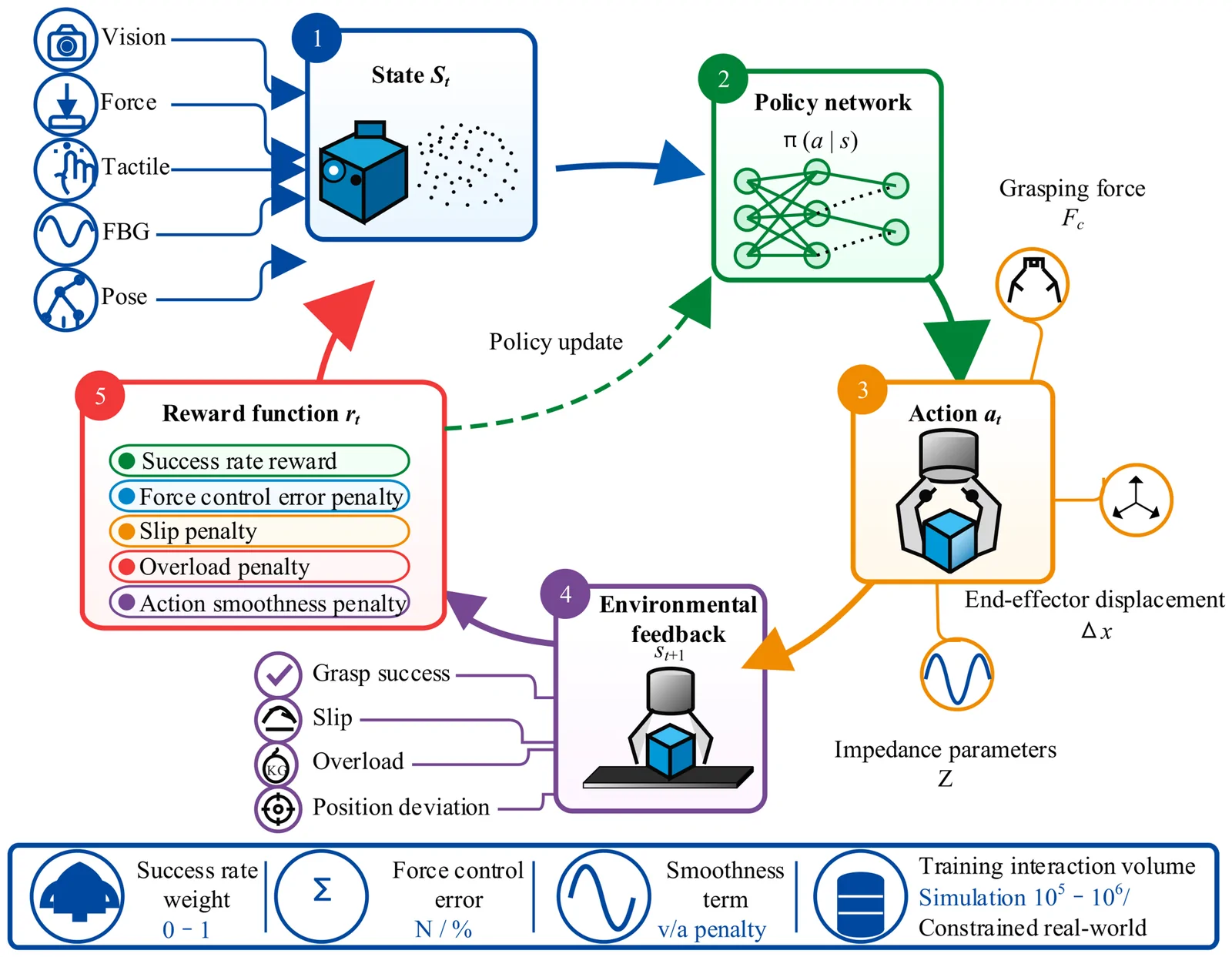
State–action–reward interaction process of reinforcement learning-based grasping.

**Figure 11:**
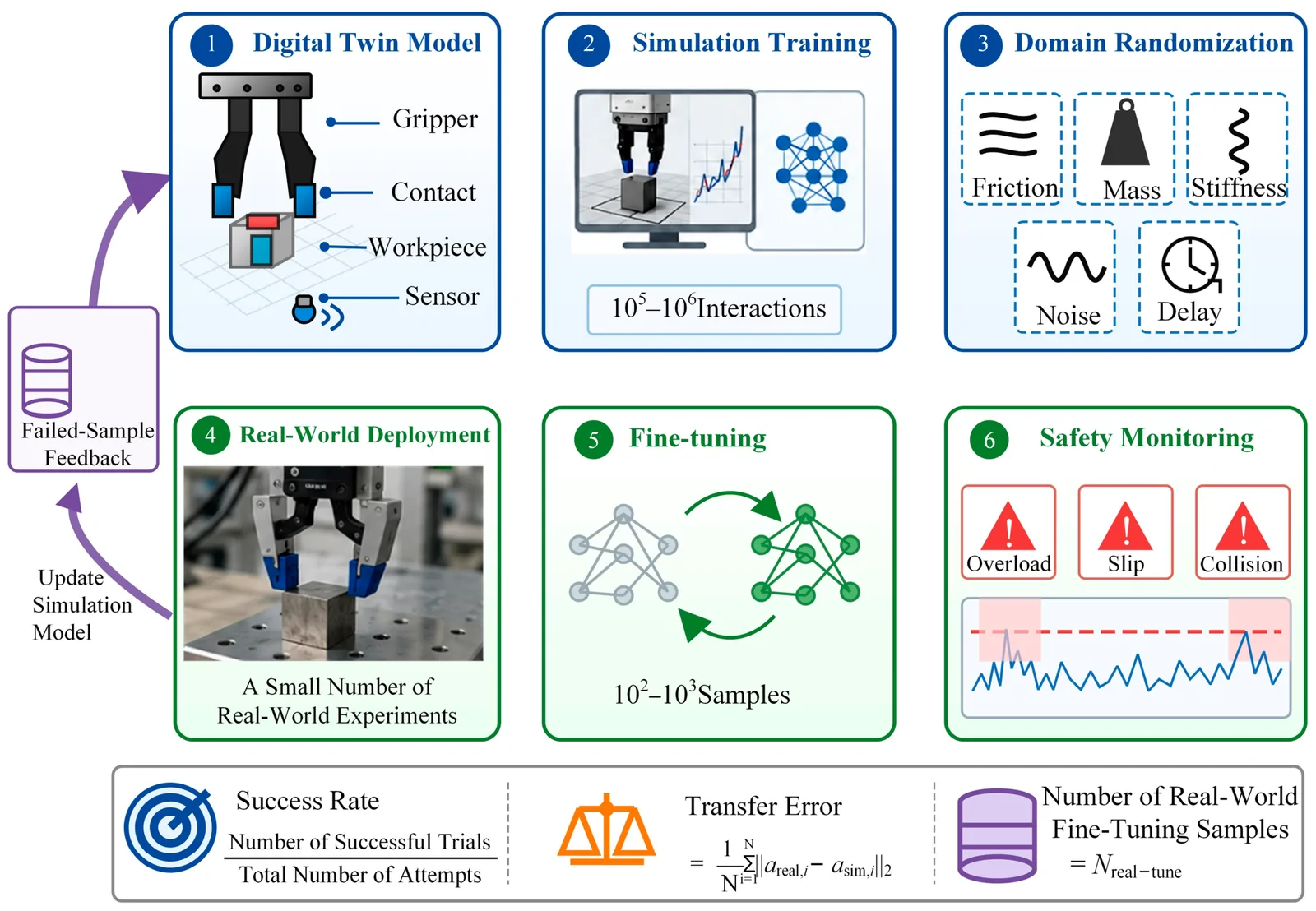
Simulation-to-real transfer framework for reinforcement learning-based grasping.

**Figure 12:**
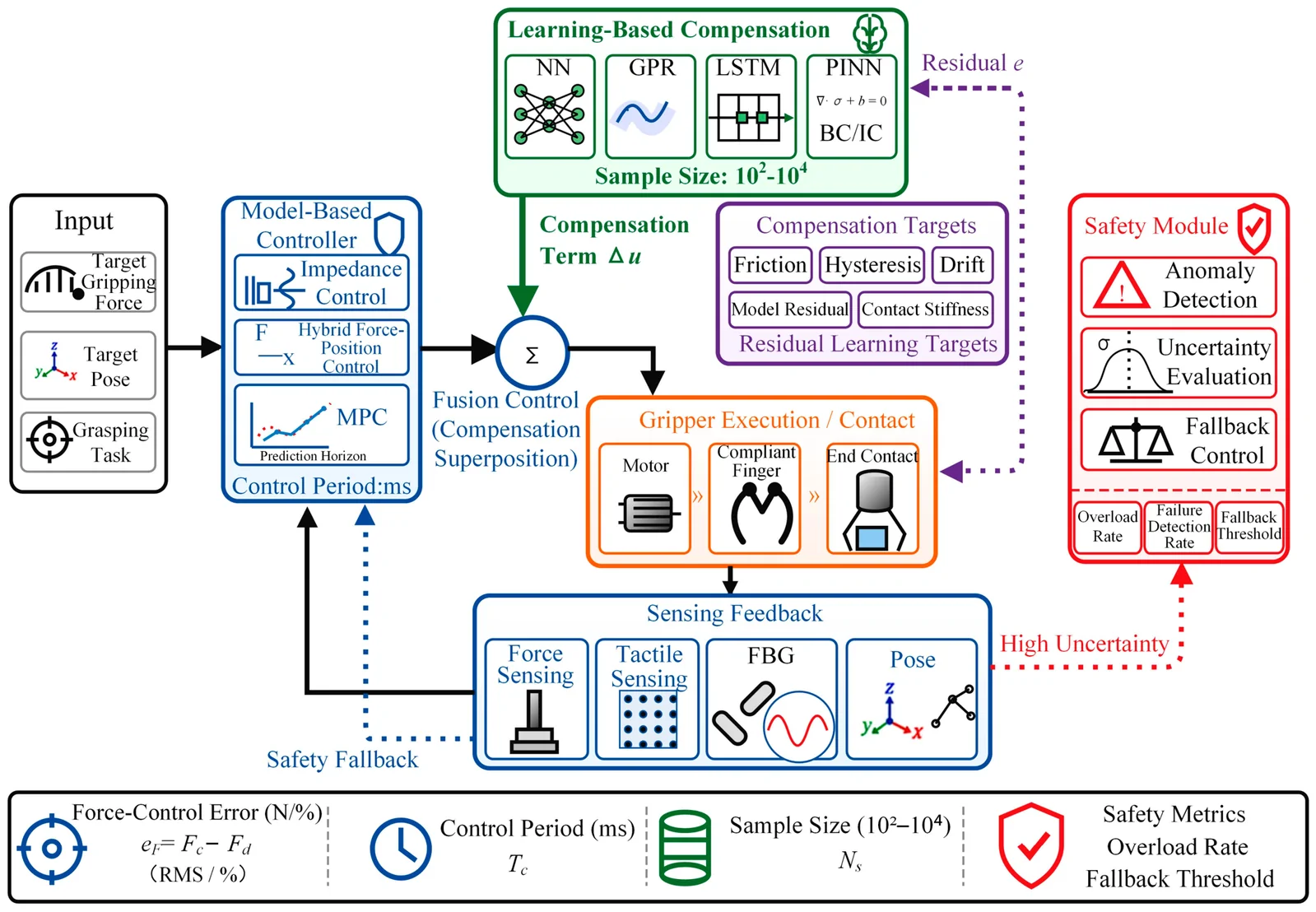
Model–data fusion architecture for grasping force control.

**Figure 13:**
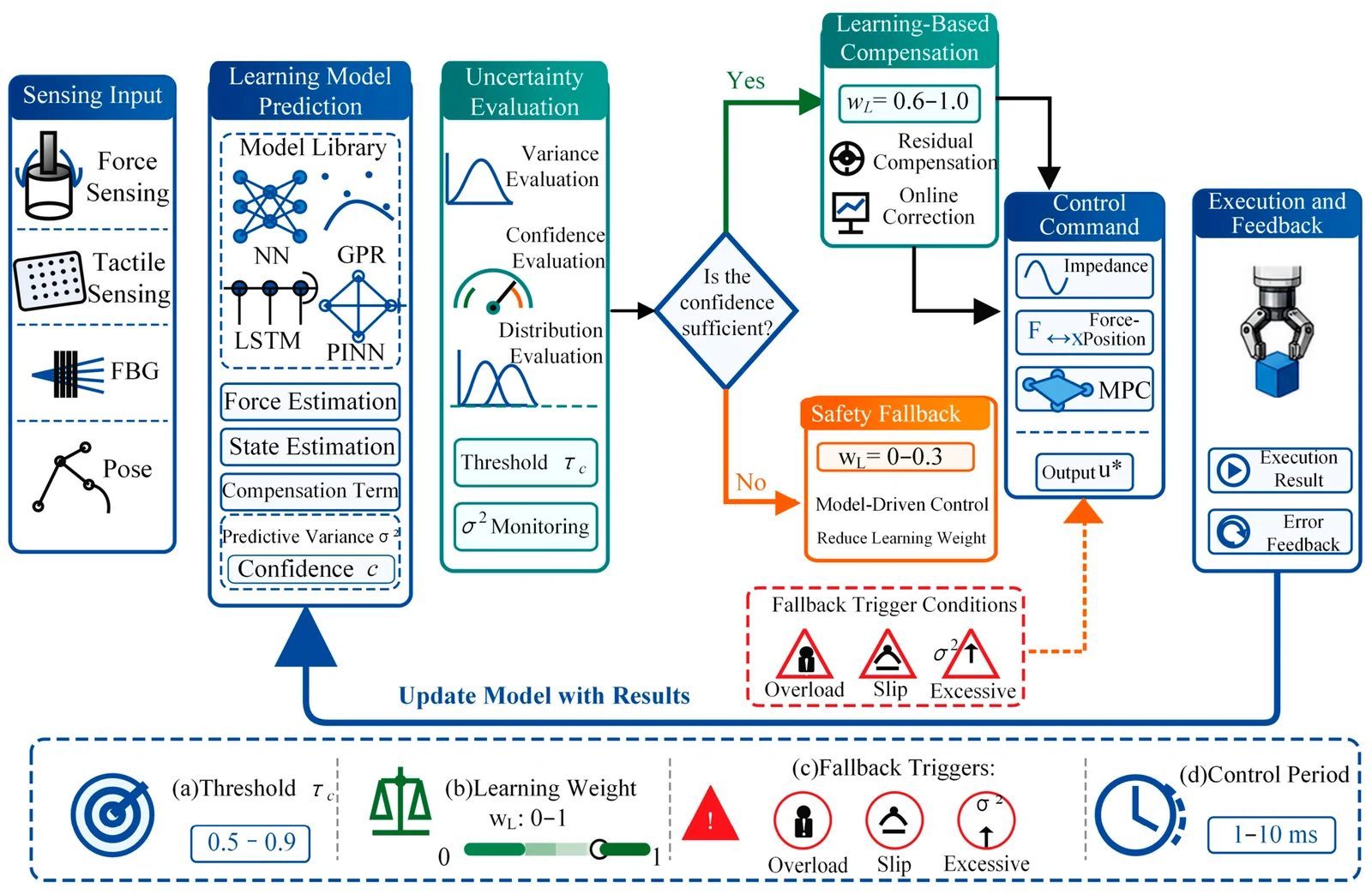
Safety fallback strategy for model–data fusion.

**Figure 14:**
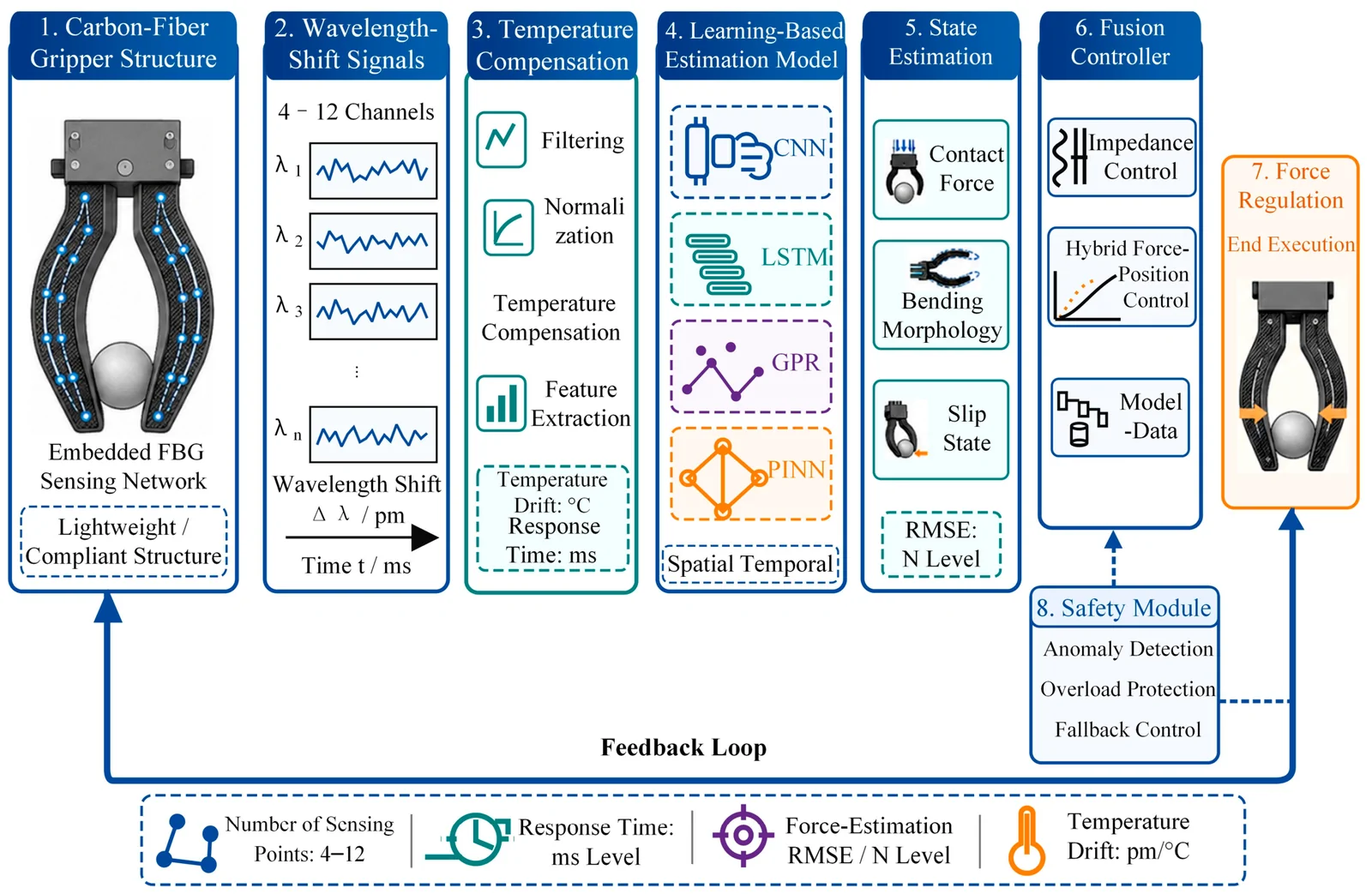
Learning-based force control mechanism of carbon fiber self-sensing grippers.

**Figure 15:**
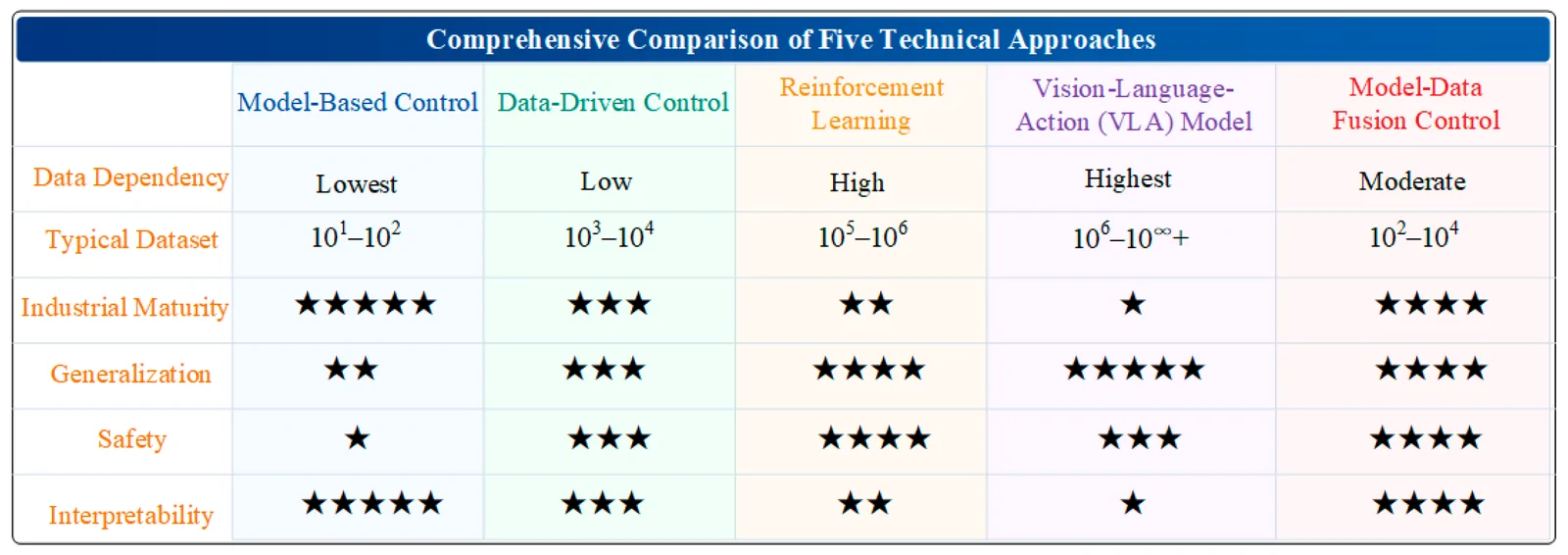
Development advantages of intelligent grasping systems.

**Figure 16:**
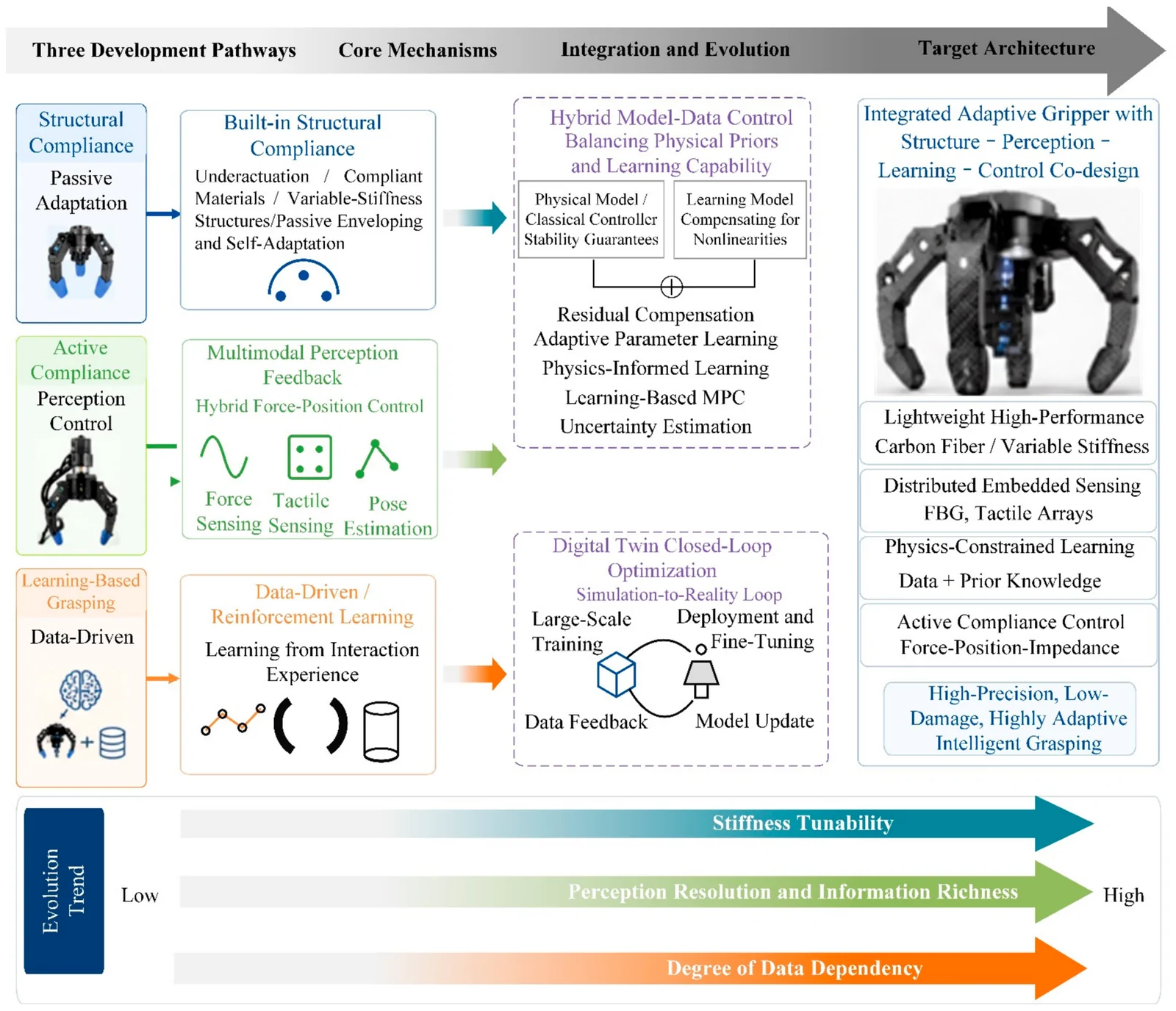
Development roadmap for adaptive robotic grippers.

**Table 1:** Comparison of three adaptive gripper routes.

Technical Route	Source of Adaptivity	Representative Advantages	Representative Limitations	Representative Application Scenarios/Operational Requirements
Structural compliance	Passive deformation of mechanisms and materials	Low cost and rapid response	Limited accuracy and online adjustability	Food handling, logistics sorting, agricultural products, and irregular/fragile object grasping where low contact damage and geometric adaptability are prioritized over high force control accuracy
Active compliance	Sensory feedback and force control strategies	Programmable force regulation and relatively high accuracy	High cost and integration complexity	Precision assembly, semiconductor handling, insertion, and fragile component manipulation requiring controlled contact force, high positioning accuracy, and stable interaction
Learning-enabled grasping	Data-driven interaction experience	Strong adaptability to complex operating conditions	High data requirements and safety validation challenges	Unstructured or multi-object manipulation, variable pose grasping, and task-level policy optimization where object properties and contact conditions are uncertain or change frequently

**Table 2:** Performance comparison of representative underactuated gripper mechanisms.

Ref	Mechanism Type	No. of Actuators	DOFs	Load Capacity	Grasping Success Rate
Birglen et al. [1]	Linkage driven	1–2	3–15	10–50 N	90–98%
Odhner et al. [11]	Tendon–elastomer hybrid	1	15	15 N	93%
Catalano et al. [12]	Tendon-driven synergistic	1	19	12 N	95%
Aukes et al. [13]	Laminated elastomer	1	7	10 N	91%
Dollar et al. [9]	SDM elastomer	1	8	13 N	96%

**Table 3:** Material and performance characteristics of soft grippers.

Material	Elastic Modulus	Maximum Deformation	Typical Grasping Force	Response Time
Ecoflex 00-30 silicone	0.1 MPa	>500%	5–15 N	50–200 ms
Dragon Skin 10	0.3 MPa	>600%	8–20 N	60–250 ms
TPU (NinjaFlex)	12 MPa	300%	10–25 N	80–300 ms
Sylgard 184 silicone	2.6 MPa	200%	15–30 N	100–400 ms
Fiber-reinforced silicone	5–20 MPa	150%	20–40 N	60–200 ms

**Table 4:** Comparison of representative variable stiffness technologies.

Technology	Stiffness-Tuning Capability	Response Time	Energy Consumption	Application Scenarios
Granular jamming	Medium to high	100–500 ms	Low	Multi-shape enveloping
Interlayer interference	Medium	50–200 ms	Low	Sheet-like or flexible structures
Antagonistic tendons	Medium	30–100 ms	Low to medium	Adjustable compliant fingers
Magnetorheological or electrorheological materials	Medium to high	<50 ms	Medium	Rapid stiffness switching
Thermally induced phase-change materials	High	On the order of seconds	High	Shape locking

**Table 5:** Performance comparison of sensing methods for adaptive grippers.

Sensing Method	Accuracy	Bandwidth	Size	Disturbance Resistance
Six-axis force/torque sensor	0.05–1% F.S.	1–5 kHz	Large	Medium—inertia, gravity, and cross-axis coupling
FBG sensor	0.3–1 N	100–500 Hz	Compact and embeddable	High—EMI immunity; temperature compensation required
Piezoresistive tactile array	0.1–1 N/unit	100–500 Hz	Thin (<1 mm)	Medium—temperature drift and signal cross-talk
Capacitive tactile array	0.05–0.5 N/unit	50–200 Hz	Thin (<0.5 mm)	Low—parasitic capacitance and environmental interference
Vision/proximity sensing	0.1 mm position accuracy	30–60 Hz	Small to medium	Medium—illumination, occlusion, and surface properties
Sensorless force estimation	5–10% error	50–100 Hz	No additional hardware	Low—model uncertainty, friction, and transmission nonlinearities

Note: Disturbance resistance is evaluated according to the sensitivity of each sensing method to its dominant disturbance sources and the extent of additional compensation required. “High” indicates intrinsic resistance to most major disturbances with only limited compensation; “Medium” indicates that one or more dominant disturbances require explicit compensation; and “Low” indicates strong dependence on environmental conditions, system modeling, or disturbance compensation.

**Table 6:** Performance comparison of force control algorithms.

Control Method	Force Error	Position Error	Control Frequency
Hybrid force–position control	0.1–1 N	0.05–0.1 mm	500 Hz–1 kHz
Impedance control	0.3–1 N	0.1–0.5 mm	500 Hz–1 kHz
Admittance control	0.5–2 N	0.05–0.2 mm	500 Hz–1 kHz
Variable impedance/adaptive control	0.1–0.5 N	0.1–0.3 mm	200–500 Hz
Sliding mode control	0.1–0.5 N	0.05–0.1 mm	500 Hz–1 kHz
Robust H∞ control	0.2–0.8 N	0.05–0.2 mm	200–500 Hz
Iterative learning control	0.1–0.3 N	0.05–0.1 mm	Iterative

**Table 7:** Representative studies on carbon fiber self-sensing grippers.

Ref	Material	Key Technology	Performance Parameters	Application
Gabardi et al. [31]	CFRP laminate	Embedded FBG strain sensing	Strain measurement error < 6%	Composite structure calibration
Zhou et al. [49]	Fiber-reinforced composite	FBG birefringence-based multidirectional strain decoupling	Three-dimensional strain measurement in quasi-isotropic laminates	Anisotropic composite monitoring
Lu et al. [50]	PDMS + FBG array	Flexible sliding tactile sensing	Sensitivity 12.4 pm/N; deviation < 5 pm	Finger slip detection
Jimbo et al. [51]	Continuous CFRP + thermoplastic matrix	Integrated 3D-printed tendon-driven finger	Two-degree-of-freedom decoupled actuation	Compliant grasping and dexterous manipulation
Hartomacıoğlu et al. [52]	Short carbon fiber-reinforced polyamide	Generative design and FDM printing	Weight reduction 71%; strength improvement 12.4%	High-speed industrial handling

**Table 8:** Comparison of representative data-driven control methods.

Method	Typical Input	Typical Output	Main Advantages
MLP	Force, current, joint angle	Grasping force, compensation signal	Fast inference and simple structure
CNN	Image, tactile array, FBG array	Contact region, force distribution	Strong spatial feature extraction
LSTM	Time-series force and FBG signals	Slip state, force variation trend	Dynamic behavior modeling
Transformer	Multimodal sequential signals	Action sequence	Long-sequence and multimodal modeling
GPR	Small-sample sensing data	Force estimation with uncertainty	Uncertainty quantification
PINN	Sensing data with physical constraints	Contact force and deformation/strain state	Improved interpretability and data efficiency

**Table 9:** Comparison of representative reinforcement learning algorithms for robotic grasping.

Algorithm	Action Type	Sample Requirement	Stability	Parameter Tuning Difficulty	Typical Application
DQN	Discrete action	Medium	Medium	Medium	Grasp point selection
DDPG	Continuous action	Medium to high	Low	High	Force–position control
TD3	Continuous action	Medium to high	High	High	Stable precision control
SAC	Continuous action	Medium to high	High	Medium	Complex contact optimization
PPO	Continuous/discrete action	Medium	High	Low	General robotic control
QT-Opt	Closed-loop visual grasping	High	High	High	Large-scale visual grasping

**Table 10:** Comparison of model-driven, data-driven, and fusion control methods.

Method	Framework Level	Interpretability	Data Requirement	Safety	Environmental Adaptability	Main Failure Mode	Industrial Applicability
Model driven	Control baseline	High	Low	High	Low to medium	Parameter drift	High
Purely data driven	Learning paradigm	Low	High	Low to medium	High	Out-of-distribution failure	Medium to low
Reinforcement learning	Learning paradigm	Low to medium	High	Medium	High	Reward bias	Medium
Model–data fusion	Integration architecture	Medium to high	Medium	High	Medium to high	Compensation error	High
VLA model	System-level framework	Low to medium	Very high	To be validated	High	Weak physical constraints	Research stage

**Table 11:** Key issues and optimization directions for FBG-based self-sensing grippers.

Key Issue	Main Cause	Performance Impact	Optimization Strategy
Temperature drift	Strain–temperature coupling	Zero-point shift	Reference grating and temperature compensation
Multi-channel coupling	Composite anisotropy	Calibration errors	CNN-/PINN-based signal decoupling
Hysteresis nonlinearity	Flexible deformation and friction	Force control lag	LSTM and residual compensation
Limited training data	High experimental acquisition cost	Poor generalization	Few-shot learning and transfer learning
Uncertain safety boundary	Limited interpretability of black-box models	Overload risk	Confidence-based fallback control
Sensor embedding effects	Fiber–matrix interface coupling	Reduced stiffness and lifetime	Layup and manufacturing optimization

**Table 12:** Classification of learning models for carbon fiber self-sensing grippers.

Model	Typical Input	Typical Output	Main Advantage	Main Limitation
Static mapping	Single-point FBG signal	Contact force, bending deformation	Fast inference	Limited dynamic capability
CNN	FBG array signals	Contact force distribution	Strong spatial feature extraction	High data requirement
LSTM	Time-series FBG signals	Slip state, force variation trend	Dynamic behavior modeling	Slow training convergence
GPR	Small-sample FBG data	Force estimation with confidence	Uncertainty estimation	Reduced efficiency for large datasets
PINN	FBG signals with mechanical constraints	Contact force and deformation/strain state	High interpretability	Complex constraint construction

## Data Availability

The data that support the findings of this study are available from the corresponding author upon reasonable request.
